# Advancing Amino Acid Isotope‐Inferred Trophic Ecology in an Apex Predator: Insights From a Data‐Rich Killer Whale Population

**DOI:** 10.1002/ece3.74121

**Published:** 2026-08-25

**Authors:** Eve Jourdain, Melissa A. McKinney, Anaïs Remili, Clare Andvik, Carl Fagerlund, Richard Karoliussen, Katrine Borgå, Anders Ruus

**Affiliations:** ^1^ Department of Biosciences University of Oslo Oslo Norway; ^2^ Norwegian Orca Survey Andenes Norway; ^3^ Department of Natural Resource Sciences McGill University Sainte‐Anne‐de‐Bellevue Quebec Canada; ^4^ Department of Biological Sciences Simon Fraser University Vancouver Canada; ^5^ Norwegian Institute of Water Research Oslo Norway

**Keywords:** cetacean, diet variation, feeding ecology, *Orcinus orca*, skin, top predator, trophic position

## Abstract

Amino acid‐specific nitrogen isotope analysis (δ^15^N‐AA) is increasingly used to infer trophic ecology, yet remains poorly validated in marine predators. We analyzed bulk tissue δ^15^N (^15^N/^14^N) and δ^13^C (^13^C/^12^C), and δ^15^N‐AA in 105 skin samples collected from individually identified Norwegian killer whales between 2017 and 2023. Forty‐two individuals with best documented diets in 2010–2023, based on field observations and corroborated by mercury concentrations and bulk tissue δ^15^N values in skin, provided reference diet groups against which δ^15^N‐AA‐derived hypotheses were tested: *Fish‐only* (100% fish prey; *n* = 15), *Intermediate mixed* diet (~10% mammalian prey in addition to fish; *n* = 14), and *Top mixed* diet (~50% mammals, 50% fish prey; *n* = 13). Univariate and multivariate approaches identified diet as the primary driver of δ^15^N variation across individual amino acids and multivariate isotopic space, whereas sex and life stage had little effect. However, substantial inter‐individual variation remained unexplained and appeared to reflect metabolic processes. Metrics δ^15^N_ΣTrophic—ΣSource_ (δ^15^N difference between the sum of trophic amino acids and the sum of source amino acids) and isotopic difference between glutamic acid/glutamine (Glu) and phenylalanine (Phe) (δ^15^N_Glu–Phe_) successfully identified *Top mixed* diet whales but lacked the sensitivity to identify *Intermediate mixed* diet individuals, whilst δ^15^N values of Threonine showed no relationship with trophic position. Proline (Pro) and Phe used as trophic‐source pair provided the first plausible δ^15^N‐AA‐derived trophic position estimates for killer whales and performed better than bulk isotope‐derived trophic position when a priori knowledge was not available. Multivariate clustering of δ^15^N‐AA data complemented trophic position estimates by resolving finer‐scale dietary variation occurring within trophic levels.

## Graphical abstract

Amino acid‐specific nitrogen isotope analysis (d15N‐AA) is widely used to infer trophic ecology but remains poorly validated in marine predators. Using a data‐rich killer whale population, we show that diet is the primary driver of nitrogen isotope composition of individual amino acids and of the multivariate isotopic space, with little effect of sex or life stage. However, conventional trophic metrics vary in performance, may underestimate trophic position, or miss low mammal consumption. Multivariate analyses, along with d15‐AA derived trophic position estimates, most effectively resolved dietary variations, providing a robust framework for improving d15N‐AA applications in higher consumers.

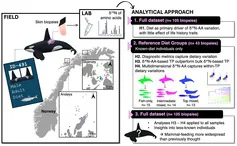

## 1 Introduction

Habitat change driven by increasing human pressures can profoundly affect apex predators such as killer whales by altering the availability, distribution, and composition of their prey resources (Sih et al. 2011; Jourdain et al. 2021). Assessing how predators respond to such changes requires detailed information on dietary variation among individuals and over time. Insights into diet and trophic plasticity are essential for understanding a population's capacity to adapt to changing environmental conditions, as well as its vulnerability to contaminant exposure and biomagnification through marine food webs (Wong and Candolin 2015). Realizing this potential, however, depends on the availability of validated dietary markers that can resolve fine‐scale differences in resource use.

In Norway, killer whales have long relied on the Norwegian Spring‐Spawning stock of Atlantic herring ( *Clupea harengus* ) as a primary prey resource (Christensen 1982; Similä et al. 1996). Historical records show dramatic fluctuations in herring biomass, from a peak of 16 million metric tonnes in the 1940s to a near collapse in the late 1960s, driven by a combination of intensive fishing and ocean‐climate variability that reduced recruitment (Dragesund et al. 1997; Sissener and Bjørndal 2005). Following this collapse, killer whales displayed markedly thinner blubber layers, suggesting nutritional stress from reduced access to their preferred prey (Christensen 1982). Stomach content analyses from the same specimens revealed diets still dominated by herring but occasionally supplemented with other prey, indicating that dietary flexibility may have helped mitigate prey scarcity (Christensen 1982). This flexibility was later confirmed in the late 1980s, when some individuals were observed alternating between herring and pinniped prey (Vongraven and Bisther 2014).

Long‐term field observations, combined with bulk tissue stable isotope analysis of skin and fatty acid profiles of blubber have since confirmed feeding plasticity as a defining trait of this killer whale population (Foote et al. 2009; Jourdain, Andvik, et al. 2020; Jourdain, Karoliussen, et al. 2020; Remili et al. 2023). Most individuals rely on herring, especially in winter when these fish are lipid‐rich and form dense schools (Jourdain, Andvik, et al. 2020; Matika et al. 2022; Slotte 1999). As herring migrate to spawning grounds, however, several killer whale groups have been observed switching seasonally to alternative fish or mammal prey, demonstrating flexibility in response to changing prey availability (Jourdain, Andvik, et al. 2020; Jourdain, Karoliussen, et al. 2020). While such behavioral plasticity can buffer whales against fluctuations in prey availability, feeding at higher trophic levels entails significant costs. Individuals that prey on pinnipeds accumulate substantially higher levels of biomagnifying contaminants, increasing their vulnerability to associated health effects (Andvik et al. 2020). Mammal predation varies widely among individuals, ranging from opportunistic or seasonal use to year‐round supplementation of the herring diet (Jourdain et al. 2017; Jourdain, Andvik, et al. 2020). These dietary shifts toward higher‐trophic‐level prey can be monitored using bulk tissue analysis of stable nitrogen isotope ratios (^15^N/^14^N, expressed as δ^15^N) of skin tissue, as δ^15^N values typically increase by ~3‰–4‰ with each trophic level (Deniro and Epstein 1981; Jourdain, Andvik, et al. 2020; Minagawa and Wada 1984). However, the detectability of such shifts depends on the relative proportions of the various prey types consumed. Indeed, bulk tissue δ^15^N reflects the weighted average isotopic composition of all prey consumed over the tissue formation and turnover period (Cerling et al. 2007). Because bulk tissue analysis measures stable isotopes in the entire sample—as a homogeneous mass rather than that of individual compounds—dietary signals from rare or opportunistic feeding on mammals can be diluted by more frequently consumed prey. As a result, occasional mammal predation may remain undetected when diets are predominantly composed of fish (Jourdain, Andvik, et al. 2020). Dietary signals can also be confounded with variations in isotopic composition at the base of the food web (hereafter *baseline variations*) which propagate through trophic levels and can rival or exceed the magnitude of trophic enrichment (de la Vega et al. 2021; McMahon and McCarthy 2016). Higher resolution approaches could overcome these limitations and more effectively track the likely intensification of shifts in killer whale foraging strategies under increasing human pressures.

Compound‐specific stable isotope analysis quantifies the isotopic composition of individual compounds within a tissue, such as δ^15^N values of amino acids (δ^15^N‐AA) in skin (McMahon and McCarthy 2016; Nash et al. 2024). Amino acids, each following distinct metabolic pathways, can be broadly categorized into *source* and *trophic* groups (Popp et al. 2007). For source amino acids, metabolic processes leave nitrogen bonds largely intact, resulting in negligible isotopic fractionation. Their δ^15^N values therefore provide a direct proxy for baseline isotopic signatures at the base of food webs (Chikaraishi et al. 2009; McClelland and Montoya 2002). In contrast, trophic amino acids are actively involved in nitrogen metabolism and undergo processes such as deamination and transamination, where amino groups are removed and recycled into the amino acid pool (Chikaraishi et al. 2007). These reactions cause substantial isotopic fractionation, leading to δ^15^N enrichment in trophic relative to source amino acids. By distinguishing between amino acids that retain baseline signatures and those that reflect trophic enrichment, δ^15^N‐AA has been posited to provide an unbiased estimation of a consumer's trophic position (Chikaraishi et al. 2009; McClelland and Montoya 2002).

Pioneering δ^15^N‐AA applications in killer whales—as in other marine mammals (e.g., Brault et al. 2019) – highlighted the value of correcting bulk δ^15^N data for baseline variation, as failing to do so may falsely suggest dietary differences that instead reflect feeding in distinct food webs (Matthews and Ferguson 2014; Troina et al. 2021). These studies also demonstrated that trophic amino acids (Glutamic acid/Glutamine *Glu*, Aspartic acid/Asparagine *Asp*, Alanine *Ala*, Isoleucine *Ile*, Leucine *Leu*, Proline *Pro*, Valine *Val*) exhibit the expected trophic enrichment patterns in killer whales (Matthews et al. 2020; Matthews and Ferguson 2014; Plint et al. 2026, in review; Troina et al. 2021). However, source amino acids such as Phenylalanine *Phe* (Matthews et al. 2020) and Lysine *Lys* (Plint et al. 2026 in review) were δ^15^N‐enriched rather than retaining baseline signatures in killer whales, a pattern also reported in other cetaceans (Matthews et al. 2020). Such variability in enrichment factors among amino acids complicates baseline corrections and has led to unrealistically low trophic position estimates in cetacean consumers (Matthews et al. 2020; Matthews and Ferguson 2014; Ruiz‐Cooley et al. 2014). Other factors such as life history traits (e.g., through differences in skin turnover rates, Thomas and Crowther 2015), dietary protein quality (McMahon, Thorrold, et al. 2015), and/or nutritional status (Lübcker et al. 2020) could be driving differential amino acid metabolism but this was not previously investigated in cetaceans. Nevertheless, the δ^15^N difference between Glu and Phe, the primary trophic‐source amino acid pairing used to account for baseline variation (Chikaraishi et al. 2007, 2009; McClelland and Montoya 2002), remains a robust indicator of differences in resource use (Matthews et al. 2021). Threonine *Thr* was identified as another promising ecological marker based on δ^15^N values approximately 8‰ lower in dentin samples of strict mammal‐specialized than in fish‐specialized killer whales from the eastern North Pacific (Matthews et al. 2021). While δ^15^N‐AA data have primarily been used to infer trophic position and habitat use, their potential to resolve finer‐scale patterns of dietary variation in cetaceans remains largely unexplored.

Here, we use a data‐rich killer whale population from Norway as a field‐validated interpretive framework to test and refine current δ^15^N‐AA‐based interpretations in trophic ecology. We examined bulk tissue δ^15^N and δ^13^C, and δ^15^N‐AA measured in 105 skin samples collected from individually identified killer whales between 2017 and 2023. Long‐term (2010–2023) records of feeding behavior (fish‐only vs. mixed diets comprising fish and mammalian prey) and life history data (sex, age) provided an independent ecological framework against which hypotheses derived from δ^15^N‐AA data could be tested. We first tested (1) the hypothesis that diet was the primary driver of δ^15^N‐AA variation, with only limited but potentially meaningful influence of life history attributes. To maximize statistical power and capture the full range of dietary, sex‐, and age‐related variation present in the population, this analysis was conducted using the complete dataset. We then focused on a subset of the most data‐rich individuals (*n* = 42), for which diets were particularly well documented in 2010–2023, to test (2) the hypothesis that previously proposed diagnostic metrics of ecological divergence (*sensu* differences in resource use) effectively captured dietary variation among individuals. Within this subset, we further hypothesized that (3) δ^15^N‐AA‐based trophic positions provide more plausible estimates relative to bulk tissue‐derived estimates. Finally, we tested (4) the hypothesis that the multidimensional nature of δ^15^N‐AA data can resolve finer‐scale dietary variation within trophic levels. Following hypothesis testing, analyses (3) and (4) were extended to the full dataset (*n* = 105) to evaluate the dietary information gained from each approach and examine the degree of concordance between methods as a validation tool.

## 2 Materials and Methods

### 2.1 Biopsy Sampling

**FIGURE 1:**
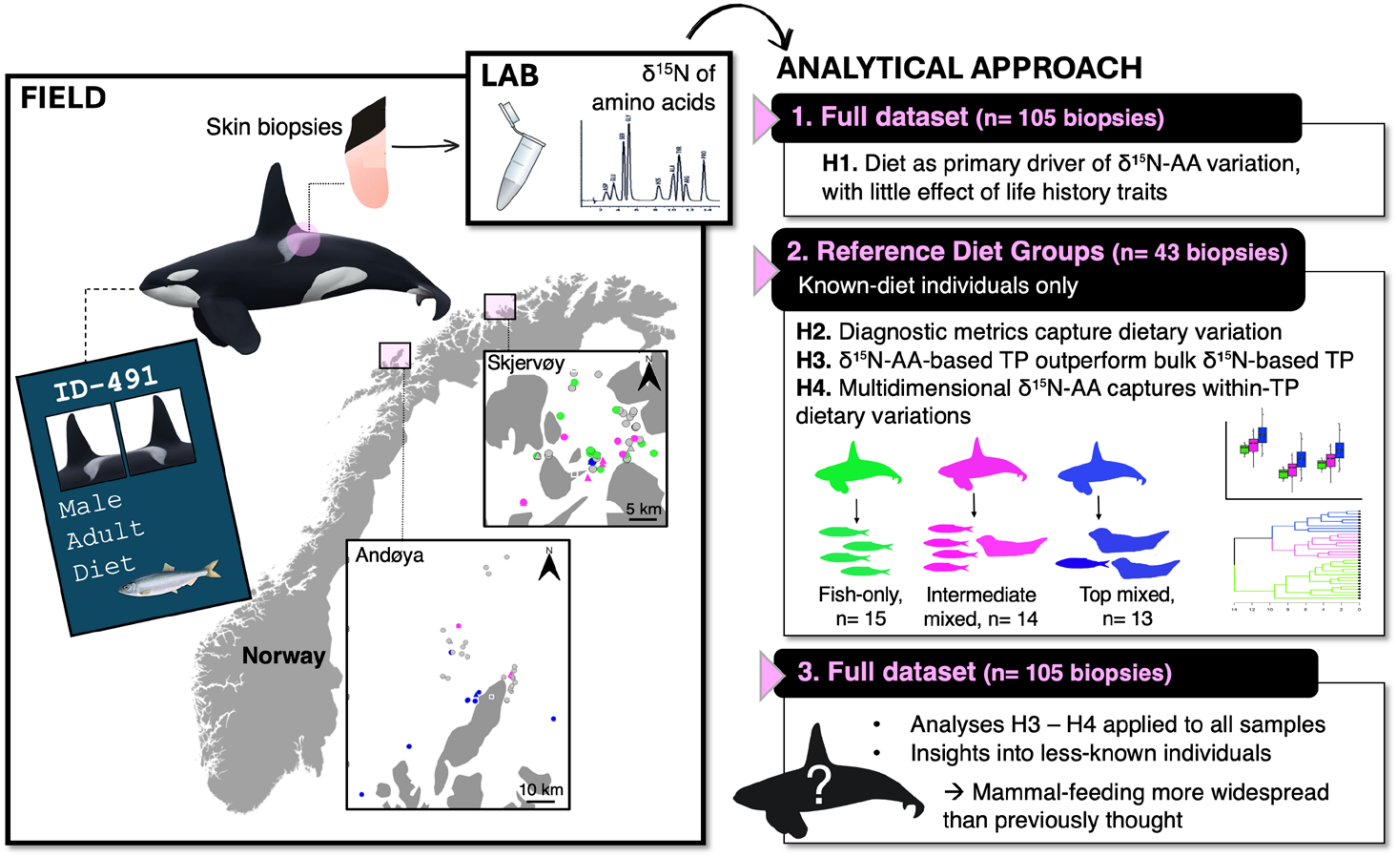
Workflow illustrating how the full dataset and a field‐validated subset of killer whales were used to test the four hypotheses of this study. (1) The full dataset (*n* = 105) was first used to test whether diet was the primary driver of δ^15^N‐AA variation, thereby evaluating the suitability of our well‐documented individuals as a field‐validated interpretive framework. Maps show the sampling locations of all individuals, illustrating that whales from all diet groups were sampled within the same geographic region and were therefore unlikely to reflect baseline isotopic differences arising from the use of different food webs. Notably, six individuals were sampled in both locations (Andøya and Skjervøy) over the course of the study and all individuals have been known to use both regions from sighting histories (Jourdain et al. 2021); (2) Following confirmation that diet was the dominant driver, a subset of 43 individuals with well‐documented diets between 2010 and 2023 was used to test hypotheses 3–4; (3) The analytical approaches validated within this reference framework were then applied to the remaining, less well‐characterized individuals as a proof of concept to assess the complementary information provided by each method and their potential to resolve dietary variation across the population. Note: Not all samples contained reliable measurements for every amino acid, as values generated from small chromatographic peaks were excluded (see Methods). Consequently, sample size varied among analyses.

Killer whale skin biopsies (*n* = 105) were collected within a 50,000 km^2^ area off northern Norway between 2017 and 2023 following previously described methods (Jourdain, Andvik, et al. 2020) (Figure 1, see Text S1 for details, Table S1). In spring (March–May, *n* = 10) and summer (June–September, *n* = 33), samples were collected off Andøya (69.329144° N, 16.137715° E) where killer whales feed on a variety of prey species including herring, lumpfish, and marine mammals (Jourdain et al. 2017; Jourdain, Karoliussen, et al. 2020). In winter (November, *n* = 62) samples were collected at herring wintering grounds, located off Skjervøy (70.032429° N, 20.996249° E) (ICES 2018), where killer whales are known to feed on the Norwegian Spring Spawning stock of Atlantic herring (Jourdain et al. 2021; Matika et al. 2022). Samples were temporarily stored onboard in a dry shipper at −196°C and, upon return onshore, stored at −80°C until analysis. Sampling was conducted under permits issued by the Norwegian Food Safety Authority (Mattilsynet FOTS‐ID: No. 10176 in 2017–2018, report no. 2016/179856; No. 18146 in 2019–2020, report no. 18/257593; and No. 24249 in 2021, report no. 20/151683). Sampled killer whales were identified from photographs using permanent, distinctive features observable on their dorsal fin and on the adjacent gray saddle patch (as introduced by Bigg 1982, see Jourdain et al. 2021 for details), and matched against a catalog available for the study population (Jourdain and Karoliussen 2021). A complementary database featuring individuals' sighting and predation histories spanning 2010–2023 was used to generate the below attribute data (central repository at the Norwegian Orca Survey).

### 2.2 Attribute Data on Sampled Killer Whales

To investigate seasonal variation in δ^15^N that could arise from dietary shifts and/or changes in nutritional status, samples were assigned a *season at sampling* reflecting prey availability at the time of collection (see Section 2.1, Table S1) (Jourdain et al. 2017; Jourdain, Karoliussen, et al. 2020). To test for potential ontogenic diet shifts (Newsome et al. 2009) and/or variations in skin turnover rates on amino acid metabolism that could arise from variations in body size (Thomas and Crowther 2015), sampled individuals' *sex* and *life stage* (reflecting age, maturity and growth) were determined using sighting histories (Table S1). *Sex* was determined using methods introduced by Bigg (1982), later refined for the study population in Jourdain et al. (2021). Sex was further confirmed via a ZFX assay on cDNA extracted from blubber samples (Han et al. 2010), conducted at Raincoast Conservation, Vancouver Canada. Age was estimated as described in Olesiuk et al. (1990, 2005) (see Text S2 for details) and was used to assign a life stage to each whale as follows: (1) *Juveniles*: ages 0–10 years (sexually and physically immature, *n* = 6), (2) *Young adults*: ages 11–20 years (sexually mature but still undergoing growth, *n* = 24), and (3) *Mature adults*: ages 21+ years (physically mature, *n* = 62). Sex and age were unknown for two and 13 individuals, respectively.

### 2.3 Tissue Analysis

#### 2.3.1 Bulk Tissue Stable Isotope Analysis

Killer whale skin samples were prepared following the protocols described in Jourdain, Andvik, et al. (2020). For each sample, bulk δ^15^N (^15^N/^14^N) were measured from a non‐lipid extracted aliquot of freeze dried and homogenized skin. In addition, bulk δ^13^C (^13^C/^12^C) values were measured from a separate lipid‐extracted aliquot because lipids are naturally depleted in ^13^C relative to proteins and carbohydrates (Post et al. 2007). If lipids remain in the sample, they can artificially lower δ^13^C values and introduce variation that reflects differences in lipid content rather than actual dietary or ecological differences. δ^13^C primarily traces the origin of carbon sources (e.g., inshore vs. offshore) and was therefore used as a proxy for feeding habitat in subsequent analyses (Hobson 1999). Elemental carbon‐to‐nitrogen (C:N) ratios were calculated from the %C and %N in the non‐lipid extracted aliquot. Analyses were conducted at the University of Oslo Stable Isotope Biochemistry Laboratory as part of a Master thesis (Fagerlund 2023). Measurements were well within established quality assurance and quality control (see Text S3 for details).

#### 2.3.2 Stable Nitrogen Isotope Analysis of Amino Acids (δ^15^N‐AA)

δ^15^N‐AA of 105 killer whale skin samples was conducted at the Ecological Change and Environmental Stressors Lab at McGill University, Canada following the protocols from Plint et al. 2026 (in review) (see Text S4 for details). Briefly, each skin sample (30–100 mg) was dried and homogenized to a fine powder. In a first step, acid hydrolysis dissociated peptide bonds and freed individual amino acids which, in a second step, were derivatized by esterification followed by N‐trifluoroacetylation (EA‐TFA) (Nash et al. 2024). An aliquot of the amino acid derivatives was then resuspended in ethyl acetate and run on the GC‐IRMS. δ^15^N values of the amino acid derivatives were measured on a Delta V Plus Isotope Ratio Mass Spectrometer (IRMS) for the following 13 individual amino acids: Alanine (Ala), Glycine (Gly), Threonine (Thr), Serine (Ser), Valine (Val), Leucine (Leu), Isoleucine (Ile), Proline (Pro), Aspartic acid/Asparagine (Asp), Glutamic acid/Glutamine (Glu), Phenylalanine (Phe), Lysine (Lys), Arginine (Arg). All samples were analyzed in duplicate. Because analytical precision decreased with smaller peak height (Figure S1), δ^15^N‐AA values generated from small peaks were discarded. Precision for measurement of selected δ^15^N‐AA values was 0.6‰ ± 0.5‰ for samples, 0.7‰ ± 0.5‰ for standards, and accuracy checked using valine (USGS74) was 2.3‰ ± 1.2‰ for checks. Upon discarding δ^15^N values generated from small peaks, the dataset was as follows: Ala: *n* = 102, Val: *n* = 78, Leu: *n* = 104, Ile: *n* = 22, Pro: *n* = 101, Asp: *n* = 101, Glu: *n* = 101, Phe: *n* = 95, Lys: *n* = 104, Thr: *n* = 94, Gly: *n* = 105, Ser: *n* = 104 and Arg: *n* = 91.

#### 2.3.3 Mercury Analysis

Total mercury (Hg), as it biomagnifies in food webs, was used as a complementary dietary marker to aid in the identification of reference diet groups (see 2.4.1). Skin Hg concentrations were analyzed by atomic absorption spectroscopy (DMA80) at the University of Oslo Toxicology lab as part of a Master thesis (Fagerlund 2023). Protocols are described in Andvik et al. (2020). Measurements were well within established quality assurance and controls (see Text S5 for details).

### 2.4 Data Treatment and Statistical Analysis

All data treatment and statistical analyses were conducted in R v. 4.2.0 for Mac (R Core Team 2018) and using *p* < 0.05 as significance level. Unless stated otherwise, statistical tests were implemented using functions available in the base R stats package. A flow chart shows how the different portions of the dataset were used in the below analyses (Figure 1).

#### 2.4.1 Identifying Reference Diet Groups

Bulk δ^15^N values and Hg concentrations (2017–2023), and predation histories (2010–2023), were used to identify isotopically distinct reference diet groups within the full set of samples (*n* = 105) (see Text S6 for details). *Fish‐only* individuals (*n* = 15) were characterized by no observational evidence of mammal predation, lowest bulk δ^15^N (mean ± SD = 11.2‰ ± 0.5‰), and lowest Hg concentrations in skin (1354 ± 515 ng/g dry weight). The *Intermediate mixed* group (*n* = 14) was observed foraging on mammal prey in 10% of encounters and had mid‐range bulk δ^15^N (11.9‰ ± 0.3‰) and Hg values (1502 ± 419 ng/g). The *Top mixed diet group* (*n* = 13) included individuals observed feeding on mammals in 50% of encounters and had the highest bulk δ^15^N (12.8‰ ± 0.5‰) and Hg values (3802 ± 954 ng/g) (Figures S2 and S3). Several samples were from the same individuals sampled repeatedly between 2017 and 2023 (four whales sampled in two different years and one whale sampled in three) (Table S2), but all samples from a given individual were consistently assigned to the same diet group. This gave us confidence that the three diet groups were robust and reflected persistent dietary habits. Statistical differences in bulk tissue δ^15^N among groups were confirmed using a Kruskal‐Wallis test, followed by pairwise Wilcoxon rank‐sum tests (Table S3, Figures S2 and S3). Because these groups represented a gradient of mammal consumption, reflected in bulk isotopic variation and independently validated through field observations, they were used as *reference* diet groups in the subsequent analyses. The remaining 63 samples, hereafter referred to as the *Default* group, are individuals with no recorded mammal‐feeding between 2010 and 2023 which could reflect either a true absence of such behavior or a failure to detect it in the field.

#### 2.4.2 Drivers of δ^15^N‐AA Variation

Benefiting from our multidisciplinary dataset, we aimed to assess the potential influence of confounding factors other than diet on δ^15^N‐AA variation. We first adopted a univariate approach to identify factors influencing each amino acid individually. We used multiple linear regression models to test the effects of diet group (*Fish‐only, Intermediate mixed*, *Top mixed, Default*), life stage (*Juveniles, Young adults, Mature adults*), sex (*Male, Female*), and sampling season (*Winter, Spring, Summer*) on the δ^15^N values of individual amino acids. Normality and homogeneity of variance of the residuals, and potential presence of influential outliers, were inspected using a Shapiro–Wilk test and/or diagnostic plots (Fox and Weisberg 2011). Repeated samples from the same individuals (Table S2) were treated as independent observations, justified by the limited number of repeat samples (11 out of 105) and the fact that all observations retained individual‐level dietary and life history attributes.

We then explored multivariate patterns of variation, in case combinations of amino acids revealed patterns not apparent in any single one, using principal component analysis (PCA) in the R package FactoMineR (Lê et al. 2008). Diet group, sex, life stage, and season at sampling were included as passive variables and projected post hoc to aid interpretation of δ^15^N‐AA variation. Significance of the variables on the response δ^15^N‐AA values was tested using a redundancy analysis (RDA) in the vegan package in R (Oksanen et al. 2022). Prior to analysis, individuals with missing data for one or several amino acids (due to discarding those δ^15^N‐AA generated from small peaks, see Section 2.3.2) were removed. PCA and RDA were therefore performed on a subset of 53 samples (including five repeat samples) with data available for all 12 amino acids (Ile not included because mostly missing data). As including or excluding repeats gave similar results (data not shown), these samples were treated as independent observations. In both univariate and multivariate linear regression, significant variables were those included in the best model, identified by forward model selection from the full to null model. Samples/individuals with unknown life stage (*n* = 13) and/or sex (*n* = 2) were discarded from the analyses.

To evaluate whether the observed δ^15^N enrichment in source amino acids (Phe and Lys; see Results) was driven by variation in baseline δ^15^N and thus, different feeding areas used by individual whales, or trophic enrichment, we examined the correlation between the sum of δ^15^N‐Phe and δ^15^N‐Lys (δ^15^N_ΣPhe,Lys_) and bulk tissue δ^15^N vs. δ^13^C values using regression analysis in R (see Text S7 for details). We further tested for potential relationship between C:N ratio and δ^15^N‐Phe and δ^15^N‐Lys to explore the effect of diet quality (protein‐ vs. lipid‐rich diets) on δ^15^N‐AA (Germain et al. 2013; McMahon, Thorrold, et al. 2015). However, because C:N ratios did not differ across diet groups, this was not considered further (see Text S7 for details, Figure S4).

#### 2.4.3 Diagnostic Metrics of Ecological Divergence

We used the subset of the most data‐rich individuals identified as reference diet groups (see Section 2.4.1, Figures S2 and S3) as a field‐validated interpretive framework to test previously proposed metrics of ecological divergence in killer whales (Matthews et al. 2021): (1) δ^15^N_ΣTrophic—ΣSource_ i.e., the difference in δ^15^N values between the sum of the trophic amino acids (Glu, Asp, Ala, Leu, Pro) and the sum of the source amino acids (Phe, Lys); and (2) δ^15^N_Glu—Phe_ i.e., the difference between δ^15^N‐Glu and δ^15^N‐Phe. We assessed differences across reference diet groups for each metric using a Kruskal‐Wallis rank sum test followed by Dunn's post hoc tests, implemented in the FSA package in R (Ogle et al. 2026). We also explored the relationship between δ^15^N‐Thr and δ^15^N_Glu—Phe_ using a correlation analysis.

#### 2.4.4 δ15N‐AA‐ and Bulk‐Derived TP Estimates

We evaluated and sought to refine previously published trophic position calculations derived from δ^15^N‐AA (TP_AA_). Five equations previously used for cetaceans were applied to the three reference diet groups (Table 1). Uncertainty in TP_AA_ estimates was quantified by propagating both analytical error (SD measured in our samples) and methodological error (SD associated with trophic enrichment factors (TEF) from the literature; listed as footnotes in Table 1). Error propagation was performed using a first‐order Taylor expansion implemented in the R package *propagate* (Ramirez et al. 2021; Spiess 2025). To evaluate the validity of TP_AA_, we calculated expected trophic positions (TP_Exp_) (equation from Pauly et al. 1998) for each reference diet group based on published diet estimates (Jourdain, Andvik, et al. 2020; see Table S4 for calculations). In addition, trophic positions derived from bulk tissue δ^15^N values (TP_Bulk_; equation from Hobson and Welch 1992) were calculated to allow direct comparison with TP_AA_ and TP_Exp_.

**TABLE 1:** Equations used to estimate trophic positions expected based on diet estimates (TP_Exp_), bulk tissue δ^15^N (TP_Bulk_), and from δ^15^N‐AA values (TP_AA_).

Equation	References
$TP\;Exp\;=\;1\;+\;\sum_{prey\;group\;i=1}^{n}\;TPi\;\times \;DCi\;/\;\sum_{i=1}^{n}DCi$	Pauly et al. 1998
TP1_Bulk_ = (2 + (δ^15^N_KW_—δ^15^N_Primary consumer_)/TEF_1_)	Hobson and Welch 1992
TP2_Bulk_ = (3 + (δ^15^N_KW_—δ^15^N_Herring_)/TEF_2_)	Hobson and Welch 1992
TP1_AA_ = (δ^15^N_KW Glu_—δ^15^N_KW Phe_—ß1)/TEF_Glu‐Phe_ + 1	Chikaraishi et al. 2007, 2009
TP2_AA_ = (δ^15^N_KW Trophic_—δ^15^N_KW Source_—ß1)/TEF_Glu‐Phe_ + 1	Chikaraishi et al. 2007, 2009
TP3_AA_ = (δ^15^N_KW Glu_—δ^15^N_KW Phe_—TEF_Glu‐Phe Seal_—ß1)/TEF_Glu‐Phe Plankton_ + 2	Germain et al. 2013
TP4_AA_ = (δ^15^N_KW Glu_—δ^15^N_KW Phe_—TEF_Glu‐Phe Plankton_—ß1)/TEF_Glu‐Phe Penguin_ + 2	McMahon, Polito, et al. 2015
TP5_AA_ = [(δ^15^N_KW Pro_—δ^15^N_KW Phe_—ß2)/TEF_3_] + 1	Rita et al. 2024

*Note:* TP_Exp_—where *i*: number of prey groups, *TPi*: trophic position for each prey group, *DCi*: relative contribution of each prey group to killer whale diet (30% and 70% as lower and upper bound, respectively, of mammal contribution based on Jourdain, Andvik, et al. 2020; see Table S4). TP1_Bulk_—where δ^15^N_KW_: bulk δ^15^N in the killer whale skin sample, δ^15^N_Primary consumer_: bulk δ^15^N measured in blue mussels *Mytilus edulis* (indicators of pelagic food web isotopic baselines; Post 2002) and collected in the same location/time period as the killer whale samples (δ^15^N_*Mussel*_: 7.27‰ ± 0.40‰, *n* = 9; data from Fagerlund 2023), TEF_1_ = 3.4‰ as mean trophic enrichment factor throughout food webs and, *2* representing the trophic level assigned to primary producers (Jardine et al. 2006; Post 2002). TP2_Bulk_—where δ^15^N_KW_: bulk δ^15^N in the killer whale skin sample, δ^15^N_herring_: bulk δ^15^N in muscle of herring prey collected following killer whale foraging events in 2020–2021 (δ^15^N_*Herring*_: 10.36‰ ± 0.96‰, *n* = 40; data from Fagerlund 2023), TEF_2_ = 1.57‰ as trophic enrichment factor estimated for delphinids (Giménez et al. 2016) and validated for dietary studies of Norwegian killer whales (Jourdain, Andvik, et al. 2020), and *3* representing the trophic level assigned to Atlantic herring (Pauly et al. 1998). TP_AA_—where δ^15^N_KW AA_: δ^15^N for a given amino acid (AA) in the killer whale skin sample; δ^15^N_KW Trophic/Source_: sum of δ^15^N for source (Phe, Lys) and trophic (Glu, Asp, Ala, Leu, Pro) amino acids in killer whale samples; the difference between δ^15^N‐Glu and δ^15^N‐Phe in primary producers ß1 = 3.4‰ (McClelland and Montoya 2002); TEF_Glu‐Phe_ and TEF_Glu‐Phe Plankton_ = 7.6‰ as single value to all trophic transfers derived from food web studies (Chikaraishi et al. 2007, 2009; McClelland and Montoya 2002); dual TEFs (accounting for lower trophic transfer in higher than primary consumers) TEF_Glu‐Phe Seal_ = 4.3‰ ± 1.2‰ (Germain et al. 2013) and TEF_Glu‐Phe Penguin_ = 3.5‰ ± 0.4‰ (McMahon, Polito, et al. 2015); all‐inclusive trophic enrichment between prey and predator for Pro minus the same calculation for Phe TEF_3_ = 4.5‰ ± 2.2‰ (Brault et al. 2019; McMahon and McCarthy 2016); and ß2 as the difference in δ^15^N values of Pro and Phe in autotrophs (3.1‰ ± 1.7‰; Chikaraishi et al. 2009).

#### 2.4.5 Fine‐Scale Dietary Resolution of δ^15^N‐AA Data

We aimed to develop a multivariate method sensitive enough to identify mixed‐diet individuals even at low levels of mammal predation. We applied a hierarchical clustering analysis to the 31 (out of 42) reference individuals with δ^15^N‐AA data available for a maximum combination of nine amino acids (Ala, Gly, Ser, Leu, Pro, Asp, Glu, Phe, Lys), after discarding Thr (insensitive to diet, see Section 3), Val, Ile and Arg (many missing data due to small peaks, see Section 2.3.2). Clustering was performed using the hclust() function in R on untransformed Euclidean distance values, appropriate for variables measured on the same scale and preserving absolute isotopic differences among individuals. Agglomerative hierarchical clustering with complete linkage was applied, to reach maximum dissimilarity among clusters and thus compact, conservative groupings. Three clusters were specified to align with our a priori classification into three reference diet groups (Figure S2b). A minimum correct assignment rate of 75% was required to validate the clustering method.

#### 2.4.6 Applications to the Full Dataset

We estimated trophic positions for all samples using the best‐performing equation TP5_AA_ (Rita et al. 2024; Table 1, see Section 3). We also applied the hierarchical clustering analysis to the 85 samples with δ^15^N‐AA available for all nine amino acids (Ala, Gly, Ser, Leu, Pro, Asp, Glu, Phe, Lys). Due to additional structure from small terminal branches, specifying *k* = 6 clusters was necessary to distinguish more meaningful clusters that diverged at larger Euclidean distances (see Section 3). We assessed whether individuals never previously observed feeding on mammals were assigned to the *Top mixed* and *Intermediate mixed* diet clusters, as an indication of previously overlooked mammal predation. We evaluated effective clustering i.e., distinctive δ^15^N‐AA profiles of the resulting clusters using a PCA in FactoMineR (Lê et al. 2008). Last, we assessed the distribution of δ^15^N‐AA‐derived trophic positions across clusters as an independent means of evaluating whether the cluster analysis effectively captured fine‐scale dietary variation. That is, we interpreted similar trophic position estimates within clusters as evidence that the multivariate δ^15^N‐AA signatures captured ecologically meaningful differences in resource use among individuals.

## 3 Results and Discussion

This study provides the most comprehensive dataset to date on compound‐specific stable isotope analysis of amino acids in a high‐level marine predator. This work is novel in its multidisciplinary approach, integrating long‐term feeding and life history data to test and refine current interpretations of δ^15^N‐AA. While several results align with patterns reported in previous studies, others challenge established findings, including those from earlier work on killer whales. This study is the first to demonstrate that the multidimensional nature of δ^15^N‐AA data can resolve dietary variation beyond trophic position estimates, expanding the potential applications of amino acid isotope ecology.

### 3.1 Drivers of δ^15^N‐AA Variation

**FIGURE 2:**
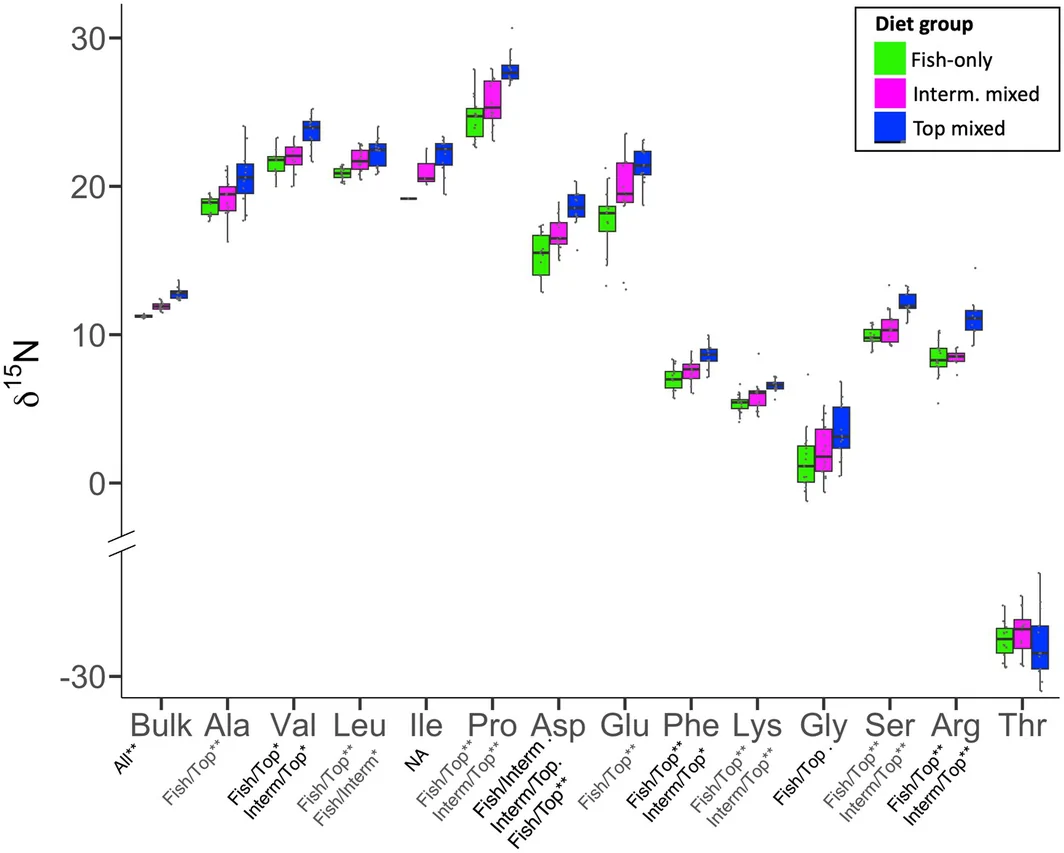
Bulk tissue δ^15^N (far‐left) and δ^15^N‐AA values (in ‰) measured in the skin of the most data‐rich killer whales, organized by reference diet group, sampled in Norway between 2017 and 2023. Box summarizes second and third quartiles, horizontal line inside the box depicts the median, whiskers show the first and fourth quartiles. Each data point represents one sample. Data points outside the box and whiskers are outliers. Significance levels for pairwise Dunn's post hoc tests that followed Kruskal–Wallis rank sum test are shown along the x‐axis with significance levels denoted as: *p* < 0 ‘***’, *p* < 0.001 ‘**’, *p* < 0.01 ‘*’, *p* < 0.05 ‘.’ Difference across diet groups was not possible to assess for Ile due to only one sample for the *Fish‐only* group.

Trophic amino acids (Ala, Glu, Asp, Ile, Leu, Pro and Val) had higher δ^15^N‐AA values (21.05 ± 2.69, range: 12.86‰–30.67‰) than bulk tissue δ^15^N (11.82 ± 0.51, range: 10.87‰–13.67‰) and source amino acids (Phe, Lys) (6.95 ± 1.40, range: 4.11‰–12.36‰) (Figure 2, Table S3). A trophic–source offset of 14‰ is consistent with established baselines (Friscourt et al. 2024; Matthews et al. 2020; Matthews, Yarnes, et al. 2024; Troina et al. 2021). Across reference diet groups, enrichment increased consistently from fish‐only whales to whales increasingly consuming mammals in addition to fish (Figure 2).

**FIGURE 3:**
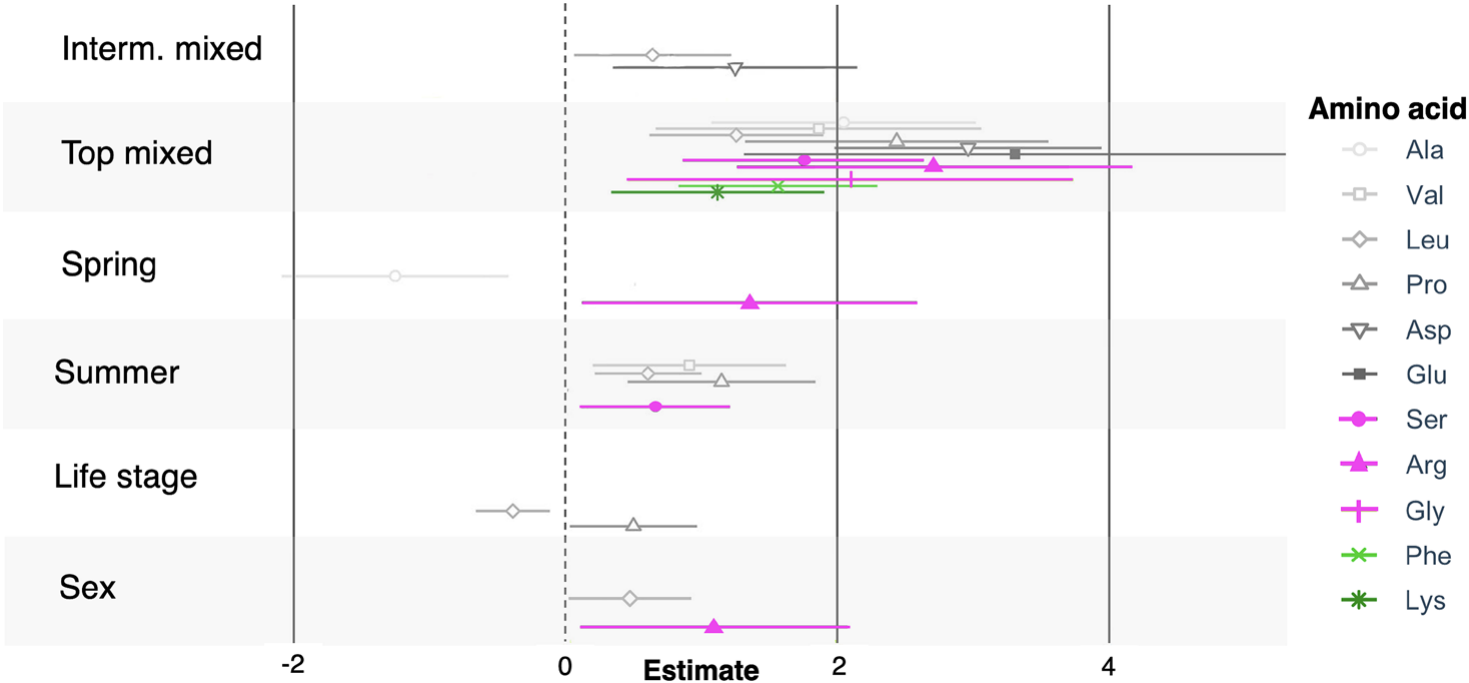
Effect sizes (coefficient estimates: Symbol and 95% confidence interval: Shaded area) estimated for each significant explanatory variable identified by the multiple linear regression models fitted to the δ^15^N values of individual amino acids. Reference levels were *Fish‐only* for the *diet* variable, *Winter* for *season at sampling* and *Female* for *sex*. See Table S5 for detailed model output. Colors are to facilitate differentiation among trophic (gray), source (green) and metabolic (pink) amino acids beyond the symbol shapes. Note that neither of the tested variable (diet, season, life stage and sex) had an effect on Thr, therefore not included in the plot.

Diet was a significant predictor of δ^15^N values for all amino acids (except for Thr), including Phe and Lys; whereby the *Top mixed* diet group (whales seen feeding on mammals in 50% of encounters in 2010–2023, Figure S3) had the largest effect size (Figure 3, Table S5). Sex and life stage had lower effects on a minority of amino acids only. Notably, we observed a slight age‐related δ^15^N depletion in Leu and an increase in Pro with age; whilst Leu and Arg were both δ^15^N‐enriched in males compared to females (Figure 3, Table S5). Elevated δ^15^N values of the source amino acids, Phe and Lys, in *Top mixed* and *Intermediate mixed* diet whales could reflect baseline isotopic variations corresponding to distinct feeding areas used by the different diet groups, or true trophic enrichment (de la Vega et al. 2021; McMahon and McCarthy 2016). We suggest the latter explanation is unlikely for two reasons. First, killer whales in Norway are known to move between sites along the Norwegian coast, and between coastal and offshore waters within hours as they switch prey, reducing the likelihood of consistent baseline effects (Jourdain et al. 2017; Vogel et al. 2024). Second, all whales co‐occurred and were sampled in the same study area, with no spatial segregation (Figure 1). Consistently, δ^15^N_ΣPhe,Lys_ did not correlate with bulk δ^13^C (proxy of inshore vs. offshore habitat; Hobson 1999) and only explained 11% of the bulk δ^15^N variation (Figure S5); far below the 91% reported by Matthews and Ferguson (2014) for whales sampled in distinct isotopic food webs. This modest contribution is more plausibly interpreted as limited trophic enrichment of source amino acids, as reported for other high trophic marine predators including killer whales (McMahon and McCarthy 2016, Plint et al., in review, Matthews et al. 2020). Indeed, the ~2‰ enrichment in δ^15^N‐Phe observed between fish‐only whales and whales consuming approximately 50% mammalian prey in Norway (Figure 2, Table S3) corresponds closely to half of the enrichment reported between strict fish and mammal specialists in the North Pacific (Matthews et al. 2020).

**FIGURE 4:**
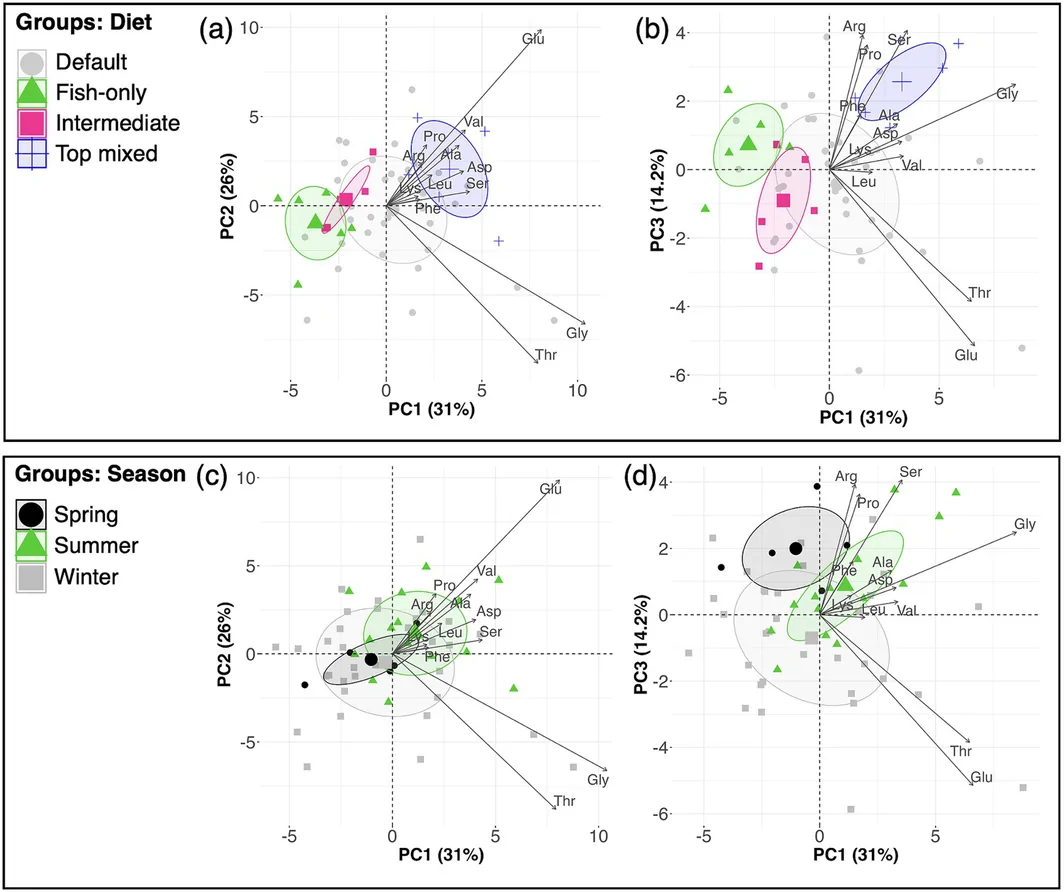
Principal component analysis of δ^15^N‐AA profiles in 53 skin samples from 48 killer whales (5 repeat samples; see Section 2.4.2). Panels show separation of (a) diet groups along PC1 vs. PC2 (b) PC1 vs. PC3, and separation of seasonal groups (season at sampling) along (c) PC1 vs. PC2 (d) and PC1 vs. PC3. Ellipses encompass 40% of the data. Redundancy analysis identified diet as the strongest effect (*F* = 4.73, *p* = 0.001) and season at sampling as a smaller but significant effect (*F* = 2.46, *p* = 0.009).

PCA further supported diet as the main driver of multivariate δ^15^N‐AA profiles while revealing additional metabolic and ecological sources of variation. Redundancy analysis showed that diet group explained most of the variation in δ^15^N‐AA profiles (RDA, *F* = 4.73, *p* = 0.001), while sampling season had a smaller but significant effect (*F* = 2.46, *p* = 0.013). Accordingly, the three reference diet groups were well separated along principal component (PC) 1 following an ecological gradient, whereby Glu, Gly, and Thr contributed the greatest variance to PC1 (31%), and Phe and Lys the lowest (Figure 4a). *Default* group individuals (never observed feeding on mammals in 2010–2023) were dispersed across the continuum, adding variation that would obscure dietary clustering without prior diet information (Figure 4a). No differentiation by sex or life stage was observed along the first two principal components (Figure S6).

In contrast, diet groups did not separate along PC2, suggesting that this axis did not reflect dietary differentiation (Figure 4a). PC2 (26%) reflected the contrast between high, positively loaded δ^15^N‐Glu, ‐Ala, ‐Val, −Leu vs. ‐Pro and low, negatively loaded δ^15^N‐Thr and ‐Gly. Gly was consistently more depleted than any source amino acid and showed the largest variation in our dataset (Figure 2, Table S3). This pattern, also reported for other cetaceans (Matthews, Smith, and Ferguson 2024; Rita et al. 2024; Troina et al. 2021), supported its unsuitability as a source amino acid (McMahon and McCarthy 2016; Nielsen et al. 2015). Furthermore, Matthews, Smith, and Ferguson (2024) reported a 10‰ difference in δ^15^N‐Gly between skin and muscle/dentin in belugas, a pattern not seen in other amino acids. Taken together, these observations support Gly as a marker of internal metabolic processes, for example, nitrogen routing and amino‐acid interconversion (reviewed and discussed in O'Connell 2017). Similarly, Thr exhibited the lowest δ^15^N values and lacked trophic enrichment (Figure 2, Table S3). Its unique catabolic pathways, involving limited transamination, suggest it reflects endogenous synthesis and catabolic processes rather than dietary input (Chikaraishi et al. 2009; Germain et al. 2013; Nielsen et al. 2015; O'Connell 2017). There was ~66% unexplained variance in the RDA, reflecting substantial individual‐level variation as well as additional unmeasured metabolic factors, as revealed in PC3.

PC3 (14% of variance) contrasted high δ^15^N‐Ser, ‐Pro, and ‐Arg with low δ^15^N‐Glu and ‐Thr, as an independent axis of isotopic variation, reflecting processes distinct from those driving the dietary and metabolic patterns observed along PC1 and PC2 (Figure 4b). Notably, the lack of differentiation of seasonal clusters along PC1 or PC2 (Figure 4c), but then apparent along PC3 (Figure 4d), suggests isotopic patterns reflective of seasonal shifts in prey composition (else than related to the mammal‐predation aspects captured in our reference diet groups) and/or metabolism. While the foraging strategies of Norwegian killer whales during spring and summer remain largely unknown, energetic balance likely differs from winter when herring are most abundant and energy‐rich (Slotte 1999). Such shifts in energetic status may alter metabolic routing and nitrogen balance, thereby influencing amino acid‐specific δ^15^N patterns (Lübcker et al. 2020). Killer whale samples collected in *summer* had higher δ^15^N values for Val, Leu, Pro, and Ser, while those collected in *spring* had lower δ^15^N‐Ala and higher δ^15^N‐Arg (Figure 3, Table S5). This pattern is consistent with δ^15^N depletion of Ala and δ^15^N enrichment of Ser and Pro reported in fasting elephant seals (Lübcker et al. 2020). Future work should combine assessments of body condition with δ^15^N‐AA measurements to better elucidate these relationships. Overall, our results supported the hypothesis that diet was the primary driver of δ^15^N‐AA variation, supporting the use of our diet groups as an appropriate framework for reassessing ecological variation metrics.

### 3.2 Diagnostic Metrics of Ecological Divergence

Both δ^15^N_ΣTrophic—ΣSource_ and δ^15^N_Glu—Phe_ were higher in the *Top mixed* diet group than in the *Fish‐only* diet group (δ^15^N_ΣTrophic—ΣSource_: 14.6‰ ± 0.6‰ vs. 13.3‰ ± 0.6‰; δ^15^N_Glu—Phe_: 12.8‰ ± 1.4‰ vs. 10.3‰ ± 2.0‰; pairwise Wilcoxon rank sum exact tests: *p* < 0.01; Figure S7a,b). These metrics, however, failed in detecting ecological differentiation between the *Fish‐only* and the *Intermediate mixed diet* groups (Figure S7a,b). Nonetheless, δ^15^N_Glu—Phe_ increased stepwise from *Fish‐only* to *Intermediate mixed* to *Top mixed* diet whales (Figure S7b), and thus with trophic position (Chikaraishi et al. 2007, 2009; McClelland and Montoya 2002). Interestingly, this pattern was reversed in North Pacific killer whale teeth, with mammal specialists δ^15^N‐enriched for Phe (~4‰) but δ^15^N‐depleted for Glu (~2‰) compared to fish specialists (Matthews et al. 2021). This discrepancy is difficult to interpret. If it were driven by isotopic baseline variation, both Phe and Glu would be expected to shift by a similar magnitude (Lorrain et al. 2015). Moreover, Glu and Phe enrichment patterns are generally consistent between dentin and skin in other cetaceans, suggesting that tissue type is unlikely to explain the observed trends (Matthews et al. 2020; Matthews, Smith, and Ferguson 2024). Consistent with our hypothesis, our results indicate that both δ^15^N_ΣTrophic—ΣSource_ and δ^15^N_Glu—Phe_ effectively captured ecological divergence in resource use among individuals, provided sufficient high trophic level prey was consumed.

Unlike Matthews et al. (2021), we did not find a significant relationship between δ^15^N‐Thr and δ^15^N_Glu—Phe_ (linear regression: *p* = 0.48; Figure S7c). Notably however, Matthews et al. (2021) measured δ^15^N‐Thr in killer whale teeth and not skin. Thr was previously shown to differ isotopically among tissues (e.g., skin/muscle; see Matthews, Smith, and Ferguson 2024), pointing to different enrichment dynamics owing to possible differences in tissue‐specific routing of Thr and making direct comparison between the two studies uncertain. Our results are also in contrast with other studies reporting δ^15^N‐Thr values decreasing systematically with each trophic transfer (Bradley et al. 2015; Germain et al. 2013). A weak but not significant trend of δ^15^N‐Thr against δ^15^N_Glu‐Phe_ (Figure S7c) could be due to limited dietary contrasts in our data due to much dietary overlap (see Jourdain, Andvik, et al. 2020). The δ^15^N‐Thr data could also reflect individual variation in metabolism and dietary protein content, as supported by high loadings of Thr along PC1(diet), PC2 (metabolism) and PC3 (season; Figure 4) (Fuller and Petzke 2017; Peters and Harper 1985).

### 3.3 δ15N‐AA‐ and Bulk‐Derived TP Estimates

**FIGURE 5:**
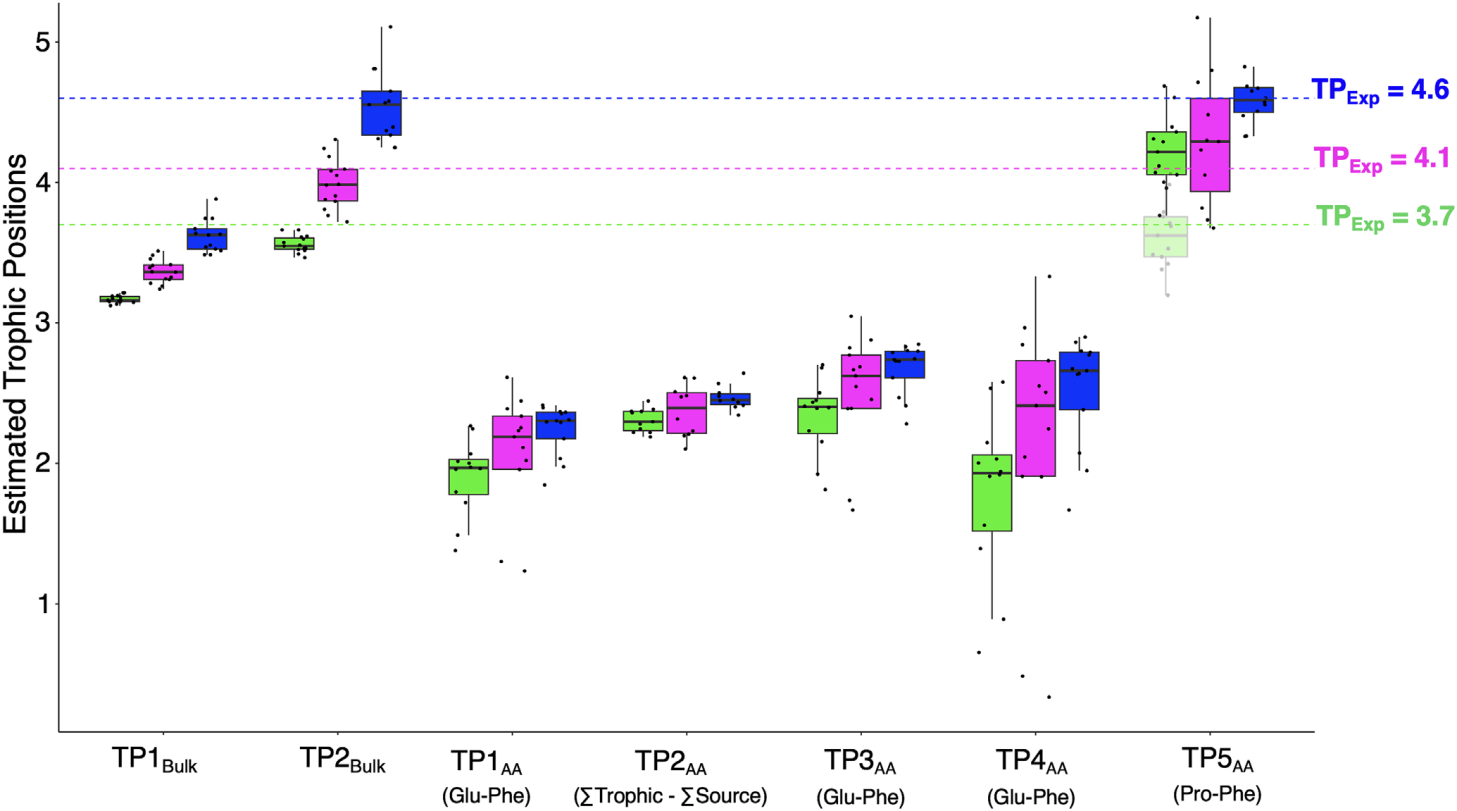
Trophic positions estimated from bulk tissue δ^15^N (TP_Bulk_) and from δ^15^N‐AA (TP_AA_) using the equations listed in Table 1. Expected trophic positions (TP_Exp_) for the three reference diet groups are shown as dashed lines i.e., *Fish‐only*: Green, *Intermediate mixed*: Pink, *Top mixed* diet: Blue. Box summarizes second and third quartiles, horizontal line inside the box depicts the median, whiskers show the first and fourth quartiles. Each data point represents one sample. The transparent green boxplot overlaid on the TP5_AA_ distributions illustrates improved agreement with expected trophic position (TP_Exp_) for the *Fish‐only* group when applying a higher trophic enrichment factor (5.5 vs. 4.5) to this diet group.

Expected trophic positions (TP_Exp_) of well‐known individuals were 3.7 for the *Fish‐only*, 4.1 for the *Intermediate mixed*, and 4.6 for the *Top mixed* diet groups (Pauly et al. 1998; Figure 5, Table S4). Trophic positions based on bulk tissue δ^15^N applying a mean trophic enrichment factor relative to a primary consumer baseline (TP1_Bulk_), and δ^15^N‐AA using Glu‐Phe as amino acid pair or ∑_Trophic_ − ∑_Source_ (TP1‐4_AA_) all yielded estimates that were unrealistically low (Figure 5). TP2_Bulk_ using known trophic enrichment from herring to killer whale (Jourdain, Andvik, et al. 2020) aligned well with TP_Exp_ but ultimately relied on a priori knowledge of diet composition. In contrast, TP5_AA_ based on the Pro‐Phe amino acid pair aligned well with TP_Exp_ (Figure 5), despite relying solely on literature‐derived trophic enrichment factors and requiring no a priori assumptions about diet. In this regard, our hypothesis that δ^15^N‐AA methods provide more plausible trophic position estimates than bulk tissue analyses was supported.

Notably though, TP5_AA_ slightly overestimated TP for the *Fish‐only* group (Figure 5). Increasing the trophic enrichment factor (TEF) for *Fish‐only* diet whales from 4.5‰ (as computed in TP5_AA_, Table 1) to 5.5‰ substantially improved agreement (3.6 ± 0.2 vs. 4.2 ± 0.3) with TP_Exp_ = 3.7 (Figure 5). A higher TEF for these whales could be justified if their diet resulted in a greater protein‐lipid imbalance compared to whales consuming a lipid‐rich mixed diet that includes mammals; indeed, individuals feeding on diets less similar to their own tissues are expected to exhibit less direct dietary routing, resulting in higher TEFs (McMahon, Thorrold, et al. 2015; Nuche‐Pascual et al. 2018). However, having to assign individual‐ (or species‐) specific TEFs would defeat the purpose of δ^15^N‐AA, since consumers' diets are typically unknown a priori. In fact, uncertainty in TEF values accounted for the largest uncertainty in TP estimates (Table S6). Refining these parameters should therefore remain a priority for future studies (Nielsen et al. 2015; Ramirez et al. 2021).

Rita et al. (2024) reported plausible trophic position estimates for fin whales using the Pro‐Phe pair, in contrast to earlier studies that used Glu‐Phe and yielded implausible estimates in cetaceans (Matthews and Ferguson 2014; Matthews et al. 2020; Ruiz‐Cooley et al. 2014). Pro may be better suited for two reasons. One possible explanation is that Pro is highly abundant in collagen‐rich skin tissues (Albaugh et al. 2017), potentially resulting in improved analytical precision relative to Glu. In our dataset, Pro showed the strongest δ^15^N enrichment with increasing mammal consumption (Figure 2, Table S3), a pattern also reported in other studies (Matthews, Yarnes, et al. 2024; Rita et al. 2024). Secondly, Pro is less sensitive to diet quality than Glu (Chikaraishi et al. 2015; Fuller and Petzke 2017; McMahon, Thorrold, et al. 2015), resulting in stronger and more consistent enrichment per trophic step, which could contribute to more reliable trophic position estimates.

### 3.4 Fine‐Scale Dietary Resolution of δ^15^N‐AA Data

**FIGURE 6:**
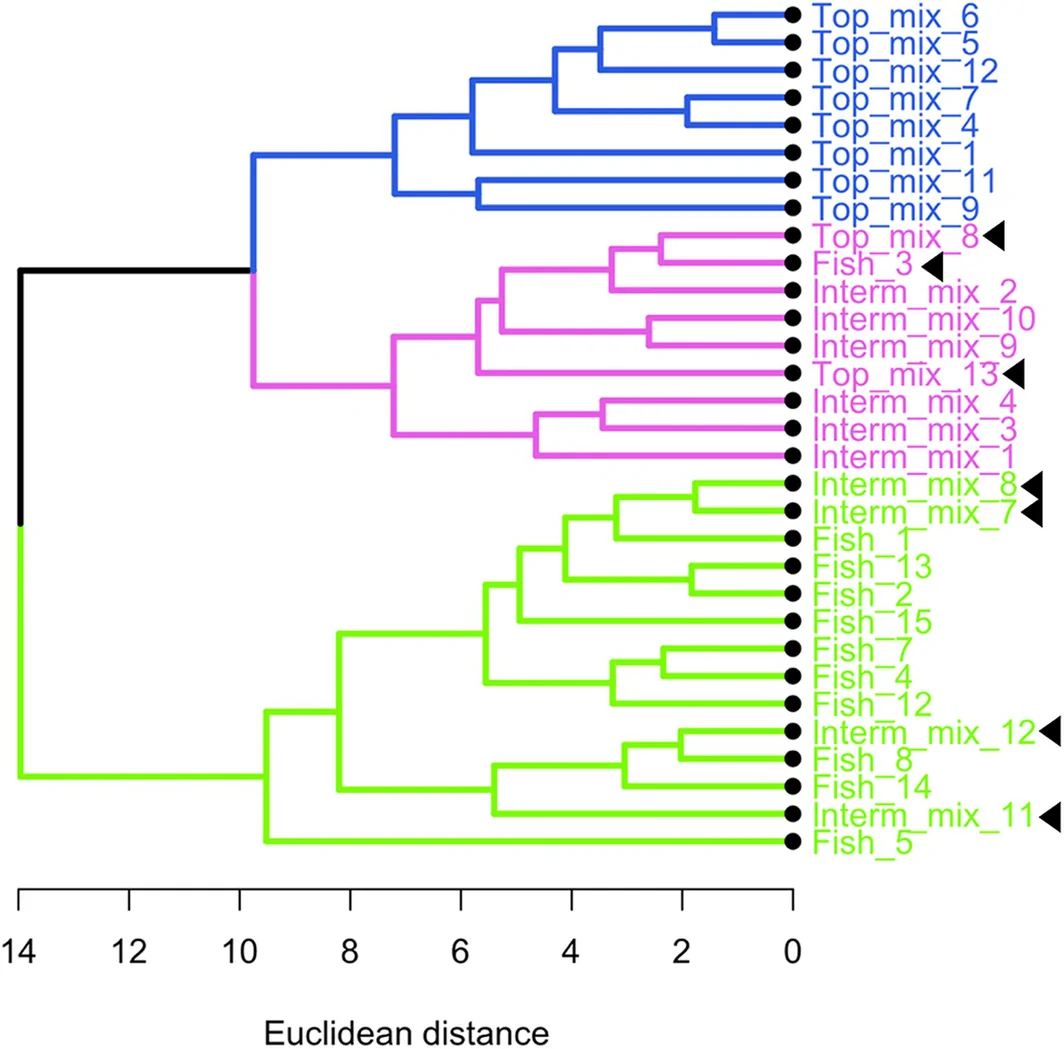
Hierarchical clustering of the 31 most data‐rich killer whales for which δ^15^N values were available for the nine amino acids Ala, Gly, Ser, Leu, Pro, Asp, Glu, Phe and Lys. Clustering was performed using Euclidean distance and complete linkage. Colors indicate the three resulting clusters which, based on assigned samples, were identified as *Top mixed* (blue), *Intermediate mixed* (pink) and *Fish‐only* (green) diet groups. Black arrows indicate the 7 samples (22%) assigned to unexpected clusters (but see Section 3.4).

The hierarchical clustering analysis performed on the 31 model samples with δ^15^N available for a maximized combination of nine amino acids correctly assigned 24 individuals to the expected *Fish‐only* vs *(Top and Intermediate) mixed* diet clusters (78% success) (Figure 6). The remaining 22% (unexpected assignments) may or may not represent misclassifications. The four whales assigned to the *Fish‐only* diet cluster while being in fact from the *Intermediate mixed* diet group were only rarely observed preying on mammals (proportions of encounters observed foraging on mammals in 2010–2023 = 0.1 ± 0.1, Figure S3d), suggesting mammal consumption was low, seasonal, or opportunistic. Because these whales were sampled in winter during peak herring abundance (Jourdain et al. 2021), mammal predation may have been negligible or nonexistent during the tissue integration window (i.e., about 50% replacement of nitrogen in bottlenose dolphin skin in 48 ± 19 days; Giménez et al. 2016); in which case their isotopic profiles correctly aligned with a fish‐only diet. Similarly, the unexpected assignment of samples *Top mix_8* and *Top mix_13* to the *Intermediate mixed* diet group may reflect minimal consumption of mammal prey within the tissue integration window. The assignment of sample *Fish_3* to the *Intermediate mixed* diet cluster while being in fact from the *Fish‐only* diet group probably reflect missed observations of predation events. The method's true accuracy may therefore be considered higher. If anything, the method is conservative and likely underestimates mammal predation due to limits imposed by tissue integration periods and sampling biases. Uncertainty in cluster assignment may also arise from methodological choices inherent to the clustering approach, including the clustering algorithm and distance metric selected.

Our clustering framework capitalizes on the multivariate structure of δ^15^N‐AA data combined with amino acid‐specific TEFs that provide complementary diagnostic power. For example, differences in δ^15^N between the *Fish‐only* and *Top mixed* diet groups were significant for all amino acids (except for non‐tested Ile and Thr; Table S3, Figure 2) but only Leu and Asp could identify subtle mammal consumption (i.e., distinguished *Intermediate mixed* diet individuals from the *Fish‐only* ones; Table S3, Figure 2). Leu and Asp undergo substantial transamination and deamination during trophic transfer yet, are among the few amino acids relatively insensitive to dietary protein content and metabolic routing; thus, exhibiting stable fractionation across diets (Fuller and Petzke 2017). As a result, variation in their δ^15^N values is more likely to reflect diet rather than metabolic processes, making them particularly robust indicators of mammal predation and key variables for teasing out subtle dietary differences in the present cluster analysis.

### 3.5 Applications to the Full Dataset

**FIGURE 7:**
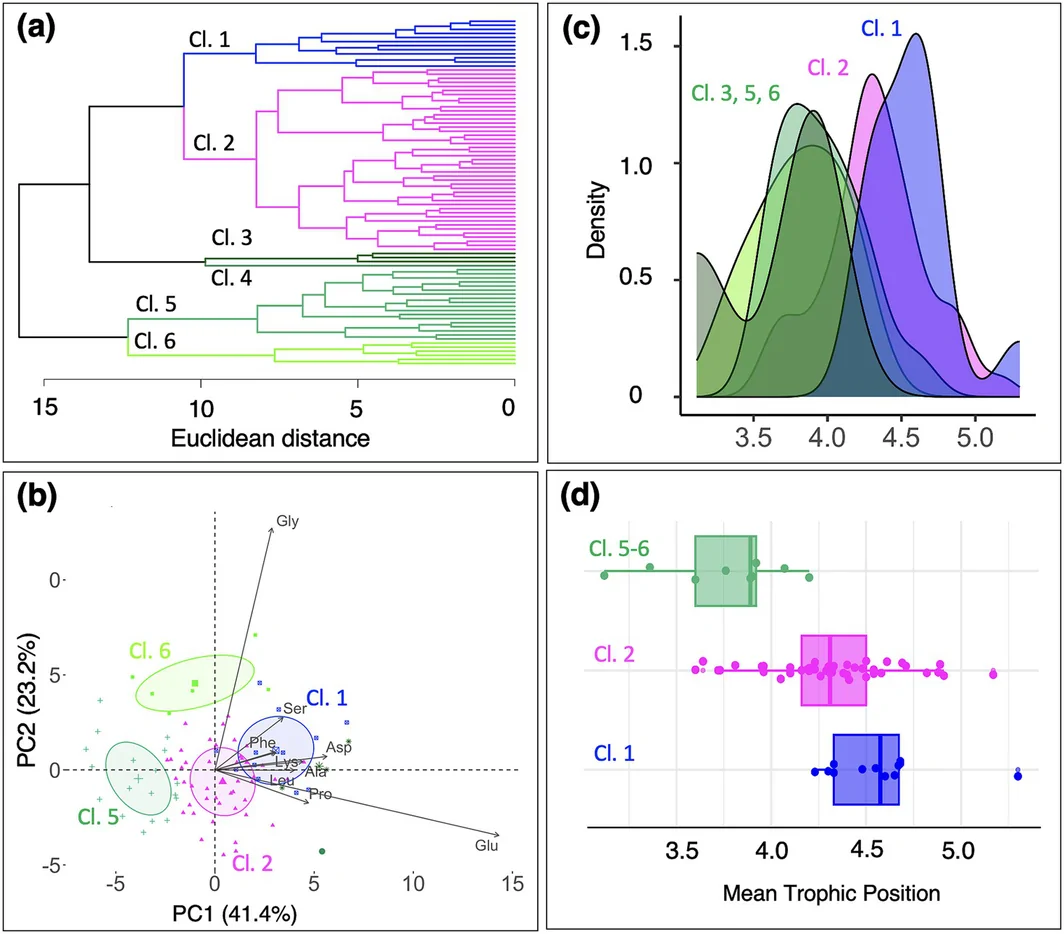
(a) Hierarchical clustering of the 85 killer whales for which δ^15^N values were available for the nine amino acids Ala, Gly, Ser, Leu, Pro, Asp, Glu, Phe, and Lys. Clustering was performed using Euclidean distance and complete linkage. Due to additional structure from small terminal branches (Clusters 3 and 4), specifying *k* = 6 was necessary to distinguish more meaningful clusters (Clusters 5 and 6) that diverged at larger Euclidean distances. Based on sample assignments, clusters are as follows: Cluster 1: *Top mixed*, Cluster 2: *Intermediate mixed*, Cluster 3 and 4: Positioned at intermediate distances between the *Mixed* diet and *Fish‐only* groups, with unclear classification, Cluster 5 and 6: *Fish‐only*. (b) Principal component analysis of the six clusters showing clear separation into largely non‐overlapping groups. Because Clusters 3 and 4 only contained three and one individual, respectively, ellipses containing 40% of the data could not be drawn. (c) Density distributions of mean trophic position by cluster, with y‐axis showing relative density and colors indicating cluster membership—except for Cluster 4 which only contained one individual. (d) Boxplots of TP_Pro‐Phe_ estimated using the best performing equation (TP5_AA_ from Rita et al. 2024; see Section 3) for each identified cluster. Box summarizes second and third quartiles, vertical line inside the box depicts the median, whiskers show the first and fourth quartiles, and data points represent individual samples.

The *Default* samples (less data‐rich individuals) revealed a continuum of amino acid profiles from *Fish‐only* to *Top mixed* diet whales, suggesting that more individuals feed on mammals than observed (Figure 4a,b). Accordingly, the clustering method assigned three and 34 of the *Default* samples to the *Top mixed* (Cluster 1, identified based on reference individuals assigned to this cluster) and *Intermediate mixed* (Cluster 2) diet clusters, respectively (Figure 7a). Cluster 1 contained all (except one) reference *Top mixed* diet whales, regardless of being sampled in spring, summer, or winter. *Top mixed* diet whales were consistently observed preying on pinnipeds over multiple years, suggesting a degree of specialization (Jourdain et al. 2017; Vongraven and Bisther 2014; Figure S3d). This nearshore foraging requires intentional habitat use and specific hunting behaviors to successfully target pinnipeds (Jourdain et al. 2017; Vogel et al. 2024). By comparison, *Intermediate mixed* diet whales known from field observations were typically observed capturing porpoises opportunistically, often in brief and inconspicuous events (EJ unpublished data), making such behavior far less likely to be recorded. Isotopic evidence suggests low‐level or opportunistic mammal predation supplementing a primarily fish‐based diet may be common in Norwegian killer whales, also evidenced by quantitative fatty acid signature analysis (QFASA) in blubber (Remili et al. 2023). Non‐overlapping δ^15^N‐AA profiles for the six inferred clusters (Figure 7b) confirmed the effectiveness of the clustering method and highlighted its high discriminatory power, potentially exceeding the level of classification (mammal‐ vs. fish consumption) applied in this study. Whether these distinct clusters correspond to finer variations in diets (e.g., different fish prey) is an intriguing question that warrants further investigation.

Trophic position estimates derived from δ^15^N‐AA (TP5_AA_) provided additional validation of the clustering method (Figure 7c). TP5_AA_ for Cluster 1 (4.6 ± 0.3, *n* = 12) matched exactly TP_Exp_ = 4.6 estimated for the *Top mixed* diet group (Figure 5, Table S4). TP5_AA_ for Cluster 2 (4.3 ± 0.3, *n* = 45) spanned the full range of TP5_AA_ estimates across the dataset, with a mean that coincided well with TP_Exp_ = 4.1 for the *Intermediate mixed* diet group. Most distant (Euclidean distance) Clusters 3–6 had the lowest (combined) mean TP5_AA_ (3.9 ± 0.3, *n* = 24) close to the TP_Exp_ = 3.7 estimated for *Fish‐only* whales. Examining trophic position by season at sampling suggested a possible increase in TP5_AA_ from winter to summer (Figure S8), providing ecological context and additional confidence in the dietary structure identified in our analyses. Indeed, it is ecologically plausible that killer whales feed at higher trophic levels during spring and summer, reflecting a shift from winter specialization on abundant, lipid‐rich herring to a more generalist diet that includes occasional mammal prey. Consistent with our hypothesis, our results confirm that the multidimensional nature of δ^15^N‐AA data can resolve dietary variation beyond trophic position estimates, providing a powerful framework to quantify inter‐individual differences along ecological gradients.

## 4 Conclusion

This study presents the largest δ^15^N‐AA dataset yet assembled for an apex predator and the first to integrate individual‐level information on diet and life history, providing an independent ecological framework against which δ^15^N‐AA‐based interpretations of trophic ecology could be tested. Diet emerged as the primary driver of both univariate and multivariate δ^15^N‐AA variation, with only minor influences of life‐history traits. However, substantial inter‐individual variation remained unexplained and appeared to reflect metabolic processes, suggesting promising future applications of δ^15^N‐AA data as indicators of physiological state beyond diet in cetaceans. Our findings add to growing evidence that the source amino acids Phe and Lys are δ^15^N‐enriched in killer whales rather than retaining baseline isotopic values. We therefore recommend interpreting these amino acids alongside bulk δ^15^N and δ^13^C measurements to distinguish trophic enrichment from baseline variation associated with differences in feeding areas. Previously proposed metrics of ecological divergence, δ^15^N_ΣTrophic−ΣSource_ and δ^15^N_Glu−Phe_, successfully identified whales with substantial mammal consumption but lacked the sensitivity to resolve intermediate dietary strategies; while δ^15^N‐Thr showed no relationship with trophic position. The Pro‐Phe (instead of Glu‐Phe pair) provided the first plausible δ^15^N‐AA‐derived trophic position estimates for killer whales, though trophic position alone was insufficient to distinguish fish specialists from individuals consuming moderate amounts of mammalian prey. These approaches are therefore well suited to detecting major shifts in trophic level but are less informative for finer‐scale dietary variation that occurs within trophic levels. We show that exploiting the multidimensional nature of δ^15^N‐AA data within a complementary clustering framework can quantify inter‐individual dietary variation even when prey switching has little or no effect on trophic position. This opens new opportunities to investigate trophic ecology in cetacean populations undergoing shifts in prey resources driven by climate change and other anthropogenic pressures, even when these changes occur without marked changes in trophic level.

## Author Contributions

**Eve Jourdain:** conceptualization (equal), data curation (lead), formal analysis (lead), funding acquisition (equal), investigation (lead), methodology (equal), project administration (equal), resources (equal), visualization (lead), writing – original draft (lead), writing – review and editing (lead). **Melissa A. McKinney:** investigation (equal), methodology (equal), project administration (equal), resources (equal), writing – review and editing (equal). **Anaïs Remili:** investigation (equal), methodology (equal), resources (equal), writing – review and editing (equal). **Clare Andvik:** data curation (equal), funding acquisition (equal), investigation (equal), resources (equal), writing – review and editing (equal). **Carl Fagerlund:** investigation (equal), resources (equal), writing – review and editing (equal). **Richard Karoliussen:** data curation (equal), funding acquisition (equal), resources (equal), writing – review and editing (equal). **Katrine Borgå:** funding acquisition (equal), investigation (equal), project administration (lead), writing – review and editing (equal). **Anders Ruus:** funding acquisition (equal), investigation (equal), project administration (lead), writing – review and editing (equal).

## Funding

This work was supported by Norges Forskningsråd, project 315392.

## Conflicts of Interest

The authors declare no conflicts of interest.

## Supporting information


**Text S1:** Supplementary methods on biopsy sampling.
**Text S2:** Supplementary methods on killer whale aging method.
**Text S3:** Supplementary methods on bulk stable isotope analysis.
**Text S4:** Supplementary methods on stable nitrogen isotope analysis of amino acids (δ^15^N‐AA).
**Text S5:** Supplementary methods on mercury (Hg) analysis.
**Text S6:** Supplementary methods on identifying reference diet groups.
**Text S7:** Supplementary methods on assessing influence of variability in isotopic baseline and diet quality.
**Table S1:** Summary of the 105 killer whale skin samples collected in Norway between 2017 and 2023 and included in the analysis of stable nitrogen isotope analysis of bulk tissue and amino acids (δ^15^N‐AA), where: *Season* (at sampling, W: winter, SS: spring–summer), Sex (M: male, F: female, NA: unknown), Life stage (1: 0–10 years, 2: 11–20 years, 3: 21+ years) and *reference diet group* (Fish‐only: fish‐specialists, Interm.: individuals observed foraging on mammalian prey in 10% of encounters in 2010–2023, and Top: individuals observed foraging on mammals in 50% of encounters; Text S6).
**Table S2:** Details of the individuals, as well as first and following sampling date(s) of the 10 killer whales that were sampled twice, or more, between 2017 and 2023 in northern Norway.
**Table S3:** Summary that is, mean ± SD *(min–max)* of bulk tissue δ^15^N and δ^13^C (in ‰), and AA‐δ^15^N values, measured in killer whale skin for each reference diet group (Fish‐only: *n* = 15, Intermediate mixed: *n* = 14, Top mixed: *n* = 13); and results from tests assessing significant statistical difference across groups. Abbreviation ‘*Fish’* stands for *Fish‐only* in the far‐right column.
**Table S4:** Expected trophic positions (TP_Exp_) for each reference diet group were estimated from the dietary compositions reported by Jourdain, Karoliussen, et al. (2020) using Equation [2] in Pauly et al. (1998). The *Fish‐only* diet group was assumed to consume a 100% herring diet, whereas the *Intermediate mixed* and *Top mixed* diet groups were assigned pinniped contributions of 30% and 70%, respectively, corresponding to the lower and upper bounds of estimated pinniped intake for seal‐eating killer whales in Jourdain, Karoliussen, et al. (2020). Prey trophic positions followed Pauly et al. (1998), with primary producers assigned TP = 1.0, herring TP = 2.7, and pinnipeds TP = 4.0.
**Table S5:** Best model and effect sizes (coefficient estimate and 95% confidence interval CI) for the significant variables as estimated by linear multiple regression models fitted to the δ^15^N data for individual amino acids. Tested variables include *diet* group (Fish‐only, Intermediate mixed, Top mixed and Default), *sex* (Male, Female), *life stage* (Juvenile, Young adult, Mature adult), and *season at sampling* (Spring, Summer, Winter). Sample size (*N*) for each amino acid is also shown.
**Table S6:** Mean, standard deviation (SD) and 95% confidence interval (CI) of the trophic position estimates derived from δ^15^N amino acid data (TP_AA_) using the best performing equation (TP5_AA_, from Rita et al. 2024). Uncertainty in TP_AA_ estimates was quantified by propagating both analytical error (SD measured in our samples) and methodological error (SD associated with trophic enrichment factor values from the literature; listed in Table 1). Error propagation was performed using a first‐order Taylor expansion implemented in the R package *propagate* (Ramirez et al. 2021; Spiess 2025). Results obtained by propagating analytical error alone and by propagating both analytical and methodological errors are shown.
**Figure S1:** Analytical precision (difference between two repeated measurements of a same killer whale sample) in relation with peak voltage (height, in mV) as observed in our dataset, leading to discarding δ^15^N‐AA values generated from peaks < 70 mV.
**Figure S2:** (a) Bulk δ^15^N values for killer whales never observed feeding on mammal prey between 2010 and 2023 (*Fish‐only*) and for individuals observed feeding on mammals at least in one encounter (*Mixed*). (b) Bulk δ^15^N values for the three reference diet groups (see Results) used as a field‐validated interpretive framework against which δ^15^N‐AA‐derived hypotheses were tested. In both panels, boxplots show the interquartile range (second and third quartiles), with the horizontal line indicating the median and whiskers representing the first and fourth quartiles. Dots represent individual samples. Stars indicate samples assigned to unexpected clusters in the hierarchical clustering analysis (see Results).
**Figure S3:** Results of the cluster analysis performed on the 27 killer whales observed foraging on mammal prey in addition to fish (mixed diet whales) at least once. Clustering was based on similarities in bulk δ^15^N values and mercury (Hg) concentrations in skin, and the proportion of encounters involving mammal predation between 2010 and 2023 (MAMM index). Panel (a) shows the two most likely clusters, given the data. Panels (b), (c), and (d) illustrate distinct ecological characteristics of the resulting *Intermediate* and *Top mixed* diet groups, supporting biologically meaningful classifications. In all boxplots, boxes represent the interquartile range, the horizontal line the median, whiskers the first and fourth quartiles, and dots individual observations.
**Figure S4:** (a) Elemental carbon‐to‐nitrogen (C:N) ratios calculated from the %C and %N in the non‐lipid extracted aliquot (see Methods) calculated for each reference diet group (Kruskal‐Wallis test: χ^2^ = 1.22, df = 2, *p* = 0.54); and (b) correlation between δ^15^N‐Phe (blue line), and δ^15^N‐Lys (green line) and the C:N ratio, as tentative means to investigate potential influence of diet quality as confounder of dietary signal.
**Figure S5:** (a) Linear regression between the sum of δ^15^N‐Phe and δ^15^N‐Lys (δ^15^N_ΣPhe, Lys_) and bulk tissue δ^15^N, and (b) linear regression between δ^15^N_ΣPhe, Lys_ and bulk δ^13^C to evaluate potential baseline isotopic variations as confounder of dietary signal in our data.
**Figure S6:** Principal component analysis (PCA) of δ^15^N values of amino acids measured in killer whale skin samples. Sex (a) and life‐stage groups (b) were included as supplementary qualitative variables and projected post hoc onto the ordination for vizualization. In agreement with redundancy analysis results, no clear separation among sex or life‐stage groups was observed, and neither variable was identified as a significant driver of δ^15^N‐AA variation.
**Figure S7:** (a) Difference in δ^15^N values between the sum of trophic (Glu, Asp, Ala, Leu, Pro) amino acids and the sum of source (Phe, Lys) amino acids (δ^15^N_ΣTrophic—ΣSource_) (Kruskal‐Wallis rank sum test: χ^2^ = 7.7688, df = 2, *p*‐value = 0.02056; Dunn post hoc test: only Fish‐only versus Top mixed was significant: *p* = 0.016), and (b) δ^15^N difference between Glu and Phe (δ^15^N_Glu—Phe_) values, for the three reference diet groups (Kruskal–Wallis test: chi‐squared = 9.2977, df = 2, *p*‐value = 0.009573; Dunn posthoc test: only Fish‐only versus Top mixed was significant: *p* = 0.007). Box depicts second and third quartiles, horizontal line represents the median and whiskers represent the first and fourth quartiles. (c) Linear regression between δ^15^N‐Thr and δ^15^N_Glu—Phe_.
**Figure S8:** Density plot of (mean) trophic position derived from δ^15^N amino acid data (TP_AA_) per season at sampling, showcasing an increase in trophic position from winter to summer.

## Data Availability

All data generated by this study are publicly available in Figshare data repository at: https://figshare.com/s/3136b40a0f6b085a4162. The R code used for the analyses is available upon request.
