# Supplementary material for: Advancing Amino Acid Isotope‐Inferred Trophic Ecology in an Apex Predator: Insights From a Data‐Rich Killer Whale Population
Source: Ecol Evol. 2026 Aug 25;16(8):e74121. doi: 10.1002/ece3.74121 (PMC13504674; doi:10.1002/ece3.74121)
Supplement: Supplementary file 1 [file ECE3-16-e74121-s001.docx]

**Supplementary Material for:**

**Advancing amino acid isotope-inferred trophic ecology in an apex predator: insights from a data-rich killer whale population**

Eve Jourdain^1,2*^, Melissa A. McKinney^3^, Anaïs Remili^3,4^, Clare Andvik^1^, Carl Fagerlund^1^, Richard Karoliussen^2^, Katrine Borgå^1^, Anders Ruus^1, 5^

^1^Department of Biosciences, University of Oslo, 0316 Oslo, Norway

^2^Norwegian Orca Survey, 8480 Andenes, Norway

^3^Department of Natural Resource Sciences, McGill University, Sainte-Anne-de-Bellevue, Quebec H9X 3V9, Canada

^4^Department of Biological Sciences, Simon Fraser University, Vancouver, Canada

^5^Norwegian Institute of Water Research, 32821 Oslo, Norway

*Corresponding author: [evemj@uio.no](mailto:evemj@uio.no)

Table of Contents

[Supplementary Text on the Methods	2](#_Toc233482233)

[Text S1. Supplementary methods on biopsy sampling	2](#_Toc233482234)

[Text S2. Supplementary methods on killer whale aging method	2](#_Toc233482235)

[Text S3. Supplementary methods on bulk stable isotope analysis	3](#_Toc233482236)

[Text S4. Supplementary methods on stable nitrogen isotope analysis of amino acids (δ¹⁵N-AA)	3](#_Toc233482237)

[Text S5. Supplementary methods on mercury (Hg) analysis	4](#_Toc233482238)

[Text S6. Supplementary methods on identifying reference diet groups	5](#_Toc233482239)

[Text S7. Supplementary methods on assessing influence of variability in isotopic baseline and diet quality	6](#_Toc233482240)

[Supplementary Tables	6](#_Toc233482241)

[Supplementary Figures	12](#_Toc233482242)

# **Supplementary Text on the Methods**

## **Text S1. Supplementary methods on biopsy sampling**

Biopsy sampled were collected using a dart fitted with a stainless-steel tip (25 x 9 mm or 40 x 9 mm) and launched by a pneumatic darting system. Biopsy tips were cleaned with soapy water, boiled, sterilized with 95% ethanol, sonicated for 2x20 min in acetone, stored in acetone-rinsed foil and placed in clean plastic bags before use. Upon contact with the animal, the biopsy tip excised a piece of skin/blubber, bounced off and landed on the sea surface for recovery. The dorsal region, immediately below or posterior to the dorsal fin, of non-calf individuals was the target for sampling. Each sampled individual was photographed for further identification. Individuals were sampled only once in each field season (e.g., winter).

## **Text S2. Supplementary methods on killer whale aging method**

Age of the killer whales sampled for this study was estimated in order to generate data on individuals’ life stage (Juveniles: 0-10 y, Young adults: 11-20 y and Mature adults: 21+ y). This was based on photographs that composed these individuals’ sighting histories, following protocols developed by (Olesiuk et al., 1990, 2005). A subset of 22 sampled killer whales was also aged using DNA methylation-based age estimators (epigenetic clocks) developed for odontocetes as part of a previous study (Robeck et al., 2021). Age estimates obtained with this molecular technique aligned well with ages estimated from sighting histories ± 3 - 5 years (data not shown), which confirmed that our aging method based on sighting histories (see below) was appropriate and resulted in robust age data for all sampled whales.

*Males*

For males, the rate of development of the dorsal fin over time was used. The dorsal fin is secondary sexual characteristic in killer whales, with males typically developing a taller, distinctively erect, dorsal fin than females. The point at which the fin starts growing (‘sprouting’) coincides with the onset of sexual maturity, and typically occurs between 11 and 15 years of age in males (average 13 y) (Olesiuk et al., 1990, 2005). Once sprouting starts, the dorsal fin continues growing for 3 to 7 years, after which males reach physical maturity. Males were considered fully grown by the age of 21 years (Olesiuk et al., 1990, 2005). In this study, males that were juvenile-sized when first photo-identified were aged according to the rate of growth/change of their dorsal fin, visually assessed throughout their recorded sighting histories. Specifically, males were estimated to be 12 y when their dorsal fin became taller than that of females. For these, precision of age estimates was ± 4 years. Males that were fully grown when first photographed i.e., with no observable change/growth of the dorsal fin throughout their sighting histories, could not be aged with the same level of precision. These individuals were assigned a conservative age of 25 years. However, it is likely that some of these individuals were older since there are reports of 50+ years old males in North Pacific killer whale populations (Olesiuk et al., 1990, 2005). Precision was therefore unknown for these males.

*Females*

Females were aged based on body-size relative to surrounding adult whales at first sighting, in relation with calving histories. Females that showed an increase in body size throughout their sighting histories were assumed to have been juveniles when first seen. These were then estimated to have been 14 years old the year they gave birth to their first (observed) viable calf. In the North Pacific, killer whales typically give birth to a first viable offspring between 11 and 17 years (average 14 y) (Olesiuk et al., 1990, 2005). For these, precision of age estimates was ± 3 to 6 years. It was not possible to estimate age of females that were already physically mature (fully grown) when first photo identified. If accompanied by an offspring, the latter was considered as the female’s first viable calf, for which age was estimated based on body size relative to the female’s. The presumed mother’s age was then estimated retrospectively, assuming she was 12 y when gave birth to the calf. Precision surrounding age estimates for these whales was unknown.

## **Text S3. Supplementary methods on bulk stable isotope analysis**

Quality assurance procedure incorporated, into each run, three to nine replicates of two internal reference material: JGLUT (L-glutamic acid, δ^13^C = −13.43‰, δ^15^N = −4.34‰, Fisher Scientific) and POPPGLY (glycine, δ^13^C = −36.58‰, δ^15^N = 11.25‰, Fisher Scientific). To assess precision of measurement, we used three to nine measurements of quality assurance material JALA (alanine, δ^13^C = −20.62‰, δ^15^N = −3.16‰, Fisher Scientific). Measured analytical precision was 0.02 ± 0.09‰ and 0.02 ± 0.08‰ for δ^13^C and δ^15^N, respectively. δ^13^C was calibrated against the Vienna Pee Dee Belemnite (VPDB) scale using LSVEC (lithium carbonate, δ^13^C = -46.6‰) and NBS-19 (calcium carbonate, δ^13^C = 1.95‰), both from the International Atomic Energy Agency (Vienna, Austria). δ^15^N was calibrated against the AIR scale using USGS40 (L-glutamic acid, δ^15^N= -4.52‰) and USGS41 (L-glutamic acid, δ^15^N= 47.57‰) from the United States Geological Survey, Reston, VA, USA. In addition, every tenth sample was analyzed in triplicate to assess variability in sample and/or equipment, which resulted in standard deviations (SD) of 0.30 ± 0.3‰ for δ^15^N (0.03 to 1.00‰, n= 10 triplicates) and 0.33 ± 0.29 (0.02 to 0.81‰, n= 8 triplicates).

## **Text S4. Supplementary methods on stable nitrogen isotope analysis of amino acids (δ¹⁵N-AA)**

δ¹⁵N-AA of killer whale skin samples was conducted at the Ecological Change and Environmental Stressors Lab at McGill University, Canada. The samples were shipped from the University of Oslo, where originally stored, to McGill University in a dry shipper that kept the samples at -196 °C throughout transport. Upon arrival at the lab, each skin sample (30-100 mg) was dried at 80°C for 12h and homogenized to a fine powder. All following steps were then conducted on batches of seven killer whale samples at a time. For each batch, the first step exposed 3 mg of protein-rich dried skin sample to an acid hydrolysis (6N HCl at 110°C for 20 h) to dissociate peptide bonds and free individual amino acids (AAs). In a second step, and to optimize gas chromatographic analysis of the AAs (see below), the AAs were derivatized. This two-step procedure decreases compound polarity while increasing volatility. AA were derivatized by converting the carboxy group to a nonpolar ester in a reaction catalyzed by isopropanol acidified by acetyl chloride under heat. Subsequent acylation added an acyl group to the amine group using trifluoroacetic anhydride (TFAA), resulting in trifluoro acetyl isopropyl esters (TFA-IP; Silfer et al., 1991). This derivatization method offers a fair reaction yield (70-75%), while having fewer steps and improved chromatography relative to other methods (Nash et al., 2024).

An aliquot of the AA derivatives (in the TFAA: DCM solution, post-acylation) was resuspended in ethyl acetate and run on the GC-IRMS within 0.5 to 9 weeks (average 5 weeks) of storage at -20°C, falling within the 10 - 25 weeks period of expected stability for TFA-IP (Nash et al., 2024). In this last step, δ^15^N values of the AA derivatives were measured on a Thermo Scientific (Bremen, Germany) Trace GC 1310 coupled via a GC Isolink II to a Delta V Plus IRMS (GC-C-IRMS). Specifically, in the gas chromatograph (GC), derivatives were first separated on a Trajan Scientific SGE BPX5 column (60 m, 0.32mm i.d., 1.0 µm film thickness). The compounds were then sent to a GC Isolink II interface for combustion. Upon leaving the interface, analytes (in the form N_2_ gas) were transferred via a ConFlo IV, through an LN2 cold trap to remove CO_2_, to the Delta V Plus Isotope Ratio Mass Spectrometer (IRMS) for measurement of δ^15^N of the following individual AA: alanine (Ala), glycine (Gly), threonine (Thr), serine (Ser), valine (Val), leucine (Leu), isoleucine (Ile), proline (Pro), aspartic acid/asparagine (Asp), glutamic acid/glutamine (Glu), phenylalanine (Phe), lysine (Lys), arginine (Arg). Each batch of samples included three standards used for further data correction/verification, all subjected to the same treatments and storage conditions as the samples. A standard mixture of 14 AAs (in equimolar quantities) was used to calculate the offset (no scaling mix used) induced by derivatization and combustion for each AA in our samples; the δ^15^ N values of underivatized AAs used as reference were measured using a Thermo Flash elemental analyzer coupled to A Delta V Plus Isotope Ratio Mass Spectrometer. To check for accuracy, a valine check standard with certified δ^15^N value was also derivatized and analyzed with each sample batch. Casein was used as in-house reference material for routine precision checks. In addition, all samples and standards were spiked with an AA internal reference standard (norleucine, Nle) to check run-to-run variation. All samples were analyzed in duplicate.

Integration of chromatograms was done using software Isodat (Thermo Scientific, v 3.0) by specifying expected retention times of each AA based on previous runs of individual AA standards. Correct peak definition relative to instrument baseline, as well as drifting baselines and peak quality (symmetric shape) were manually checked for all samples and standards. δ^15^N values of individual AAs were determined from the voltage output (peak height) for each corresponding peak. Because analytical precision decreased with smaller peak height (Figure S1), δ^15^N-AA values generated from small peaks were discarded. Precision for measurement of selected δ^15^N-AA values was 0.6 ± 0.5‰ for samples, 0.7 ± 0.5‰ for standards and accuracy for valine checks was 2.3 ± 1.2‰*.*

## **Text S5. Supplementary methods on mercury (Hg) analysis**

We used about 2 mg of dried and homogenized killer whale skin for each sample to be analyzed. Quality controls were included in each run. To detect any presence of residual mercury in the instrument, each run started and ended with blanks. Certified reference materials were also included in each run to ensure analytical quality. Specifically, these were triplicates of DORM-4 fish protein and DOLT-5 dogfish liver (National Research Council of Canada, Ottawa, Canada) and a triplicate of internal reference standard (TORSK, cod liver). Reference materials were ordered from lowest to highest concentrations to prevent any carryover from more to lesser contaminated samples. In addition, every tenth sample was analyzed as a triplicate. For these, the mean was used for statistical analysis. To correct for instrument noise, mean blank concentrations were calculated from triplicate measurements after each run, and subtracted from sample concentrations. Hg measurements were at least one magnitude higher for killer samples relative to blanks. Certified reference materials were all within 10% of the reported Hg measurements. Standard deviations indicated a sample variability of 286 ng/g dry weight.

## **Text S6. Supplementary methods on identifying reference diet groups**

Out of the 105 samples included in this study, 27 were from killer whales observed foraging on mammal prey, in addition to fish, at least once in 2010-2023 (hereafter *Mixed diet* whales). The other 78 samples were from individuals only known to forage on fish prey (hereafter *Fish-only diet* whales) from field observations (Jourdain et al., 2017; Jourdain et al., 2020a; Jourdain et al., 2021)(EJ unpublished data) although occasional mammal consumption may have been missed in the field. Notably, *Mixed diet* killer whales engaged in mammal foraging to varying degrees, as captured by a mammal foraging frequency index (*MAMM*). For each whale, *MAMM* was calculated as the ratio of encounters involving mammal foraging to the total number of encounters recorded between 2010 and 2023, where an encounter is a group sighting at a given time and location. To control for varying prey acquisition rates across encounters, *MAMM* included actual predation/feeding events as well as other behavioral patterns indicative of pinniped-foraging (e.g., hugging shoreline and circling reefs or haul outs; Jourdain et al., 2017; Vongraven & Bisther, 2014). Bulk tissue δ^15^N values for the *Fish*-only and *Mixed diet* killer whales overlapped, indicating the presence of likely *Mixed diet* individuals in the *Fish-only diet* group (see Figure S2a). We used bulk stable nitrogen isotope ratio (¹⁵N/¹⁴N, expressed as δ¹⁵N), mercury (Hg) concentrations and predation histories (*MAMM*) to identify isotopically (δ¹⁵N) distinct reference diet groups that could be explained by long-term dietary habits (*MAMM*) and contaminant exposure (Hg).

We conducted a clustering analysis on the 27 *Mixed diet* killer whales. Clustering was based on similar mammal foraging frequencies (*MAMM*), bulk tissue δ¹⁵N values and Hg concentrations in skin. The *scale()* function in R (R Core Team, 2018) was used to scale and center the data before analysis. Euclidean distance was used to generate the similarity matrix for pairs of whales, and k-means was used as the clustering method. The gap statistic method, implemented in the R package *factoextra* (Kassambara & Mundt, 2020; R Core Team, 2018)*,* identified two as most likely number of clusters. The *Top mixed diet* cluster (n=13) was characterized by highest bulk δ¹⁵N (mean ± SD: 12.8 ± 0.5‰), highest Hg (3802 ± 954 ng/g dry weight) and most time observed foraging on mammal prey (*MAMM*= 0.5 ± 0.2). The *Intermediate mixed diet* cluster (n=14) was distinguished by lower δ^15^N (11.9 ± 0.3‰) and Hg (1502 ± 419 ng/g dry weight) and little time observed foraging on mammals (0.1 ± 0.2) (see Figure S3).

Clustering analysis of the 78 (*a priori*) *Fish-only* killer whales did not lead to biologically meaningful clusters (results not shown). Therefore, to identify a set of individuals that were most likely to be true fish-specialists, we used three criteria: bulk tissue δ^15^N ≤ 11.4‰ (11.2 ± 0.5, range: 11.1 - 11.4‰), multiple encounters (10 ± 8, range: 3 - 24) in at least two different years (5 ± 2, range: 2 – 10 y) to minimize the chance that mammal foraging occurred but was missed by observers, and Hg concentrations (mostly) lower (1354 ± 515, range: 944 - 1 678 ng/g dry weight) than the average for the full dataset (1653 ± 923 ng/g dry weight, n= 105). Selected threshold for δ^15^N matches expected values under a herring-specialized diet consumed in the few weeks prior sampling (Jourdain et al. 2020b). Because Hg bioaccumulates throughout an individuals’ lifetime, albeit with some level of maternal transfer (and elimination), it should reflect long-term feeding habits with lowest levels expected in *Fish-only* relative to *Mixed diet* whales (Ruus et al. 2015, Andvik et al. 2020).

## **Text S7. Supplementary methods on assessing influence of variability in isotopic baseline and diet quality**

To separate dietary signals from potential baseline variability, we examined the relationships between the sum of δ¹⁵N-Phe and δ¹⁵N-Lys (δ^15^N_ΣPhe, Lys_) for each sample and bulk δ^13^C (¹³C/¹²C) vs. bulk δ¹⁵N (¹⁵N/¹⁴N). We also tested for a relationship between δ^15^N-Phe, and δ^15^N-Lys, and the C:N ratio to infer relative dietary intake of protein- vs. lipid-rich prey types as potential source of ^15^N enrichment of Phe or Lys (see Results), else than trophic enrichment (e.g., Gaebler et al., 1966; Hoskin et al., 2001). Elemental carbon-to-nitrogen (C:N) ratios (calculated from the %C and %N in the non-lipid extracted aliquot) were used as a proxy for dietary macronutrient composition, with lower ratios indicating protein-rich, fish-dominated diets and higher ratios reflecting lipid-rich, mixed diets including both fish and mammalian prey. Although the C:N ratio in skin has not been formally validated as a direct indicator of dietary intake, our tentative approach was supported by marked differences in macronutrient composition among known prey of Norwegian killer whales, including lower lipid content in herring muscle (16%; Remili et al., 2023) compared with higher lipid content in harbor seal and harbor porpoise blubber (53% and 66%, respectively; Lee et al., in review).

# **Supplementary Tables**

**Table S1.** Summary of the 105 killer whale skin samples collected in Norway between 2017 and 2023 and included in the analysis of stable nitrogen isotope analysis of bulk tissue and amino acids (δ¹⁵N-AA), where: *Season* (at sampling, *W*: winter, *SS*: spring-summer), *Sex* (*M*: male, *F:* female, *NA*: unknown), Life stage (*1*: 0-10 y, *2*: 11-20 y, *3*: 21+y) and *reference diet group* (Fish-only: fish-specialists, Interm.: individuals observed foraging on mammalian prey in 10% of encounters in 2010-2023, and Top: individuals observed foraging on mammals in 50% of encounters; Text S6).

|  | **Season** | | **Sex** | | | **Life stage** | | | | **Reference diet group** | | |
| --- | --- | --- | --- | --- | --- | --- | --- | --- | --- | --- | --- | --- |
|  | **W** | **SS** | **M** | **F** | **NA** | **1** | **2** | **3** | **NA** | **Fish-only** | **Interm.** | **Top** |
| 2017 | 5 | 3 | 4 | 4 | 0 | 0 | 2 | 6 | 0 | 0 | 2 | 6 |
| 2018 | 0 | 3 | 1 | 2 | 0 | 0 | 1 | 2 | 0 | 0 | 0 | 3 |
| 2019 |  |  |  |  |  |  |  |  |  |  |  |  |
| 2020 | 17 | 0 | 15 | 2 | 0 | 3 | 2 | 10 | 2 | 1 | 2 | 0 |
| 2021 | 33 | 5 | 28 | 10 | 0 | 2 | 13 | 19 | 4 | 11 | 4 | 0 |
| 2022 | 7 | 23 | 23 | 6 | 1 | 0 | 5 | 18 | 7 | 3 | 4 | 3 |
| 2023 | 0 | 9 | 6 | 2 | 1 | 1 | 1 | 7 | 0 | 0 | 2 | 1 |
| **Total** | **62** | **43** | **77** | **26** | **2** | **6** | **24** | **62** | **13** | **15** | **14** | **13** |

**Table S2.** Details of the individuals, as well as first and following sampling date(s) of the 10 killer whales that were sampled twice, or more, between 2017 and 2023 in northern Norway

| **Whale** | **Diet** | **Sex** | **First sampling** | **Repeat sampling** |
| --- | --- | --- | --- | --- |
| ID6 | Top mixed | Male | 16/11/2017 | 09/07/2022 |
| ID 43 | Default | Male | 17/11/2020 | 18/11/2021 |
| ID25 | Fish-only | Male | 16/11/2020 | 15/11/2021 |
| ID64 | Default |  | 27/11/2021 | 24/07/2022 |
| ID13 | Interm. mixed | Male | 07/08/2021 | 18/11/2022 |
| ID14 | Interm. mixed | Male | 17/11/2020 | 18/11/2022  13/07/2023 |
| ID71 | Default | Male | 16/11/2020 | 2/12/2021 |
| ID17 | Interm. mixed |  | 17/11/2020 | 19/11/2021 |
| ID82 | Default | Male | 22/11/2020 | 14/07/2022 |
| ID21 | Interm. mixed | Male | 27/11/2017 | 31/07/2022 |

**Table S3**. Summary i.e., mean ± SD (min – max) of bulk tissue δ^15^N and δ^13^C (in ‰), and AA-δ^15^N values, measured in killer whale skin for each reference diet group (*Fish-only*: n= 15, *Intermediate mixed*: n= 14, *Top mixed*: n= 13); and results from tests assessing significant statistical difference across groups. Abbreviation ‘*Fish’* stands for *Fish-only* in the far-right column.

|  | Fish-only | Intermediate mixed | Top mixed | Kruskal-Wallis | Dunn’s post hoc tests |
| --- | --- | --- | --- | --- | --- |
| Bulk δ^15^N | 11.24 ± 0.1  (11.09 - 11.40) | 11.92 ± 0.3  (11.5-12.4) | 12.8 ± 0.4  (12.3 - 13.7) | chi-sq= 36.07, df = 2, P **< .001** | All: P **< .001** |
| Bulk δ^13^C | -19.44 ± 0.2  (-19.81; -19.19) | -19.6 ± 0.4  (-20.0; -18.9) | -19.2 ± 0.5  (-20.0, -18.0) | chi-sq= 5.97,  df = 2, P= 0.05 | none |
| *Trophic* |  |  |  |  |  |
| *δ^15^N* -Ala | 18.7 ± 0.6  (17.6-19.5) | 19.2 ± 1.3  (16.3-21.3) | 20.6 ± 2.0  (17.7-24.1) | chi-sq= 8.56,  df = 2, P= 0.01 | Top vs. Fish: P= 0.01 |
| *δ^15^N* -Val | 21.6 ± 0.9  (20.0-23.3) | 21.9 ± 1.1  (20.0-23.3) | 23.7 ± 1.1  (21.7-25.2) | chi-sq= 12.79, df = 2, P= 0.002 | Top vs. Fish: P= 0.003  Top vs. Interm: P= 0.016 |
| *δ^15^N* -Leu | 20.9 ± 0.4  (20.2-21.4) | 21.74 ± 0.8  (20.5-22.9) | 22.3 ± 0.9  (20.8-24.0) | chi-sq= 15.57, df = 2, **P < .001** | Top vs. Fish: P < .001  Interm vs. Fish: P= 0.015 |
| *δ^15^N* -Ile | 19.17 (n=1) | 21.06 ± 1.3  (20.13-22.54) | 22.0 ± 1.2  (19.4-23.3) | - | - |
| *δ^15^N* -Pro | 24.6 ± 1.5  (22.6 - 27.9) | 25.62 ± 1.6  (23.06-27.9) | 27.9 ± 1.1  (26.8-30.7) | chi-sq= 19.20, df = 2, **P < .001** | Top vs. Fish: P **< .001**  Top vs. Interm: P **< .001** |
| *δ^15^N* -Asp | 15.4 ± 1.4  (12.9 - 17.4) | 16.8 ± 1.2  (15.02-18.9) | 18.5 ± 1.2  (15.7-20.3) | chi-sq= 20.65, df = 2, **P < .001** | Interm vs. Fish: P= 0.05  Top vs. Interm: P= 0.017  Top vs. Fish: **P < .001** |
| *δ^15^N* -Glu | 17.7 ± 2.3  (13.3 - 21.2) | 19.4 + 2.9  (13.05-23.6) | 21.4 + 1.2  (18.7-23.1) | chi-sq= 16.17, df = 2, **P < .001** | Fish vs. Interm: P= 0.06  Interm. Vs Top: P= 0.06  Top vs. Fish: **P < .001** |
| *Source* |  |  |  |  |  |
| *δ^15^N* -Phe | 7.0 ± 0.8  (5.7 - 8.3) | 7.6 + 0.8  (6.05 - 8.9) | 8.6+0.8  (7.1-10.0) | chi-sq= 15.98, df = 2, **P < .001** | Top vs. Fish: **P < .001**  Top vs. Interm: P= 0.02 |
| *δ^15^N* -Lys | 5.4 ± 0.7  (4.1 - 6.7) | 5.9 + 1.1  (4.5-8.7) | 6.6+0.4  (5.6-7.2) | chi-sq= 18.28, df = 2, **P < .001** | Top vs. Fish: **P < .001**  Interm vs. Top: P= 0.007 |
| *Others* |  |  |  |  |  |
| *δ^15^N* -Thr | -27.6 ± 1.2  (-29.4 ; -25.22) | -27.06 + 1.6  (-29.3; -24.6) | -27.81 + 2.3  (-30.99--23.03) | chi-sq= 1.91,  df = 2, P= 0.39 | None |
| *δ^15^N* -Gly | 1.5 ± 2.1  (-1.2 ; 7.3) | 2.1 + 1.8  (-0.6, 5.2) | 3.4 + 1.9  (0.5-6.8) | chi-sq= 7.53,  df = 2, P= 0.02 | Top vs. Fish: P= 0.02 |
| *δ^15^N* -Ser | 9.9 ± 0.7  (8.8 - 10.8) | 10.5 + 1.2  (9.2-13.3) | 12.11 + 0.7  (10.78-13.30) | chi-sq= 21.68, df = 2, **P < .001** | Top vs. Fish: **P < .001**  Top vs. Interm: **P < .001** |
| *δ^15^N* -Arg | 8.4 ± 1.3  (5.4 - 10.3) | 8.4 + 0.6  (7.3- 9.1) | 11.22 + 1.5  (9.25-14.49) | chi-sq= 17.40, df = 2, **P < .001** | Top vs. Interm: **P < .001**  Top vs. Fish: **P < .001** |

**Table S4.** Expected trophic positions (TP_Exp_) for each reference diet group were estimated from the dietary compositions reported by Jourdain et al. (2020b) using Equation [2] in Pauly et al. (1998). The *Fish-only* diet group was assumed to consume a 100% herring diet, whereas the *Intermediate mixed* and *Top mixed* diet groups were assigned pinniped contributions of 30% and 70%, respectively, corresponding to the lower and upper bounds of estimated pinniped intake for seal-eating killer whales in Jourdain et al. (2020b). Prey trophic positions followed Pauly et al. (1998), with primary producers assigned TP = 1.0, herring TP = 2.7, and pinnipeds TP = 4.0.

| Reference diet group | Computed diet | Calculations | Calculated TP_Exp_ |
| --- | --- | --- | --- |
| Fish-only | 100% Herring | 1+(1 x 2.7 / 1) | 3.7 |
| Intermediate mixed | 70% Herring,  30% Pinnipeds | 1 + (0.7 x 2.7 + 0.3 x 4.0 / 0.7 + 0.3) | 4.1 |
| Top mixed | 30% Herring,  70% Pinnipeds | 1 + (0.3 x 2.7 + 0.7 x 4.0 / 0.3 + 0.7) | 4.6 |

**Table S5.** Best model and effect sizes (coefficient estimate and 95% confidence interval CI) for the significant variables as estimated by linear multiple regression models fitted to the δ^15^N data for individual amino acids. Tested variables include *diet* group (Fish-only, Intermediate mixed, Top mixed and Default), *sex* (Male, Female), *life stage* (Juvenile, Young adult, Mature adult), and *season at sampling* (Spring, Summer, Winter). Sample size (N) for each amino acid is also shown.

| AA | Best model | Effect size |
| --- | --- | --- |
| Ala  N=87 | Ala ~ Diet + Season  Adj-R^2^: 0.24  F= 6.52 (5, 81 DF), **p < .001** | Top mixed= 2.05 [95% CI: 1.08 - 3.02], p<.001  Default = 1.04 [95% CI: 0.33 - 1.75], p<.001  Spring = -1.25 [95% CI: -2.08; -0.42], p<.001 |
| Gly  N=89 | Gly ~ Sex + Diet  Adj-R^2^: 0.06  F=: 2.46 (4, 84 DF), p= 0.05 | Top mixed= 2.10 [95% CI: 0.47-3.74], p= 0.01 |
| Thr  N=81 | Thr ~ Diet  Adj-R^2^: 0.09  F= 3.50 (3, 77 DF), p= 0.02 | Default= 1.62 [95% CI: 0.27-2.97], p= 0.02 |
| Ser  N=89 | Ser ~ Diet + Season + Life stage  Adj-R^2^: 0.28  F= 6.82 (6, 82 DF), **p < .001** | Default= 0.75 [95% CI: 0.09-1.41], p= 0.03  Top mixed= 1.75 [95% CI: 0.87-2.64], p<.001  Summer= 0.66 [95% CI: 0.11-1.20], p= 0.02 |
| Val  N=67 | Val ~ Diet + Season  Adj-R^2^: 0.28  F= 6.26 (5, 61 DF), **p < .001** | Top mixed= 1.87 [95% CI: 0.67-3.07], p<.001  Summer= 0.91 [95% CI: 0.20-1.62], p=0.01 |
| Leu  N=89 | Diet + Season + Life stage + Sex  Adj-R^2^: 0.36  F= 8.19 (7, 81 DF), **p < .001** | Default= 0.90 [95% CI: 0.42-1.38], p<.001  Intermediate mixed= 0.65 [95% CI: 0.07-1.22], p= 0.03  Top mixed= 1.26 [95% CI: 0.62-1.90], p<.001  Summer= 0.60 [95% CI: 0.21-1.00], p<.001  Life stage= -0.39 [95% CI: -0.66; -0.12], p= 0.01  Sex= 0.47 [95% CI: 0.02 – 0.92], p= 0.04 |
| Pro  N=85 | Diet + Season + Sex + Life stage  Adj-R^2^: 0.45  F= 10.84 (7, 77 DF), **p < .001** | Top mixed= 2.44, [95% CI: 1.33 - 3.56], p<.001  Summer= 1.14, [95% CI: 0.45 – 1.83], p<.001  Life stage= 0.49, [95% CI: 0.03 – 0.96], p= 0.04 |
| Asp  N=86 | Diet + Season + Life stage  Adj-R^2^: 0.38  F= 9.79 (6, 79 DF), **p < .001** | Default= 1.34, [95% CI: 0.59 - 2.10], p<.001  Intermediate mixed= 1.25, [95% CI: 0.35 - 2.15], p=0.01  Top mixed= 2.97, [95% CI: 1.98 - 3.95], p<.00 |
| Glu  N=86 | Glu ~ Diet + Season + Life stage  Adj-R^2^: 0.17  F= 3.90 (6, 79 DF), **p= .001** | Default= 2.33 [95% CI: 0.80 - 3.87], p<.001  Top mixed= 3.31 [95% CI: 1.32 – 5.31], p<.001 |
| Phe  N=82 | Phe ~ Diet  Adj-R^2^: 0.20  F= 7.78 (3, 78 DF), **p < .001** | Default= 1.16 [95% CI: 0.57 - 1.75], p<.001  Top mixed= 1.57 [95% CI: 0.84 - 2.30], p<.001 |
| Lys  N=88 | Lys ~ Season + Diet + Sex  Adj-R^2^: 0.20  F= 4.75 (6, 81 DF), **p < .001** | Default= 0.74 [95% CI: 0.15 - 1.33], p= 0.01  Top mixed= 1.12 [95% CI: 0.34 - 1.91], p= 0.01 |
| Arg  N=76 | Arg ~ Diet + Sex + Season  Adj-R^2^: 0.24  F= 5.03 (6, 69 DF), **p < .001** | Top mixed= 2.72 [95% CI: 1.27 – 4.17], p<.001  Sex= 1.09 [95% CI: 0.11 – 2.07], p= 0.03  Spring= 1.36 [95% CI: 0.12 – 2.59], p= 0.03 |

**Table S6.** Mean, standard deviation (SD) and 95% confidence interval (CI) of the trophic position estimates derived from δ¹⁵N amino acid data (TP_AA_) using the best performing equation (TP5_AA_, from Rita et al., 2024). Uncertainty in TP_AA_ estimates was quantified by propagating both analytical error (SD measured in our samples) and methodological error (SD associated with trophic enrichment factor values from the literature; listed in Table 1). Error propagation was performed using a first-order Taylor expansion implemented in the R package *propagate* (Ramirez et al., 2021; Spiess, 2025). Results obtained by propagating analytical error alone and by propagating both analytical and methodological errors are shown.

|  |  | Analytical | | | | Analytical & Methodological | | | |
| --- | --- | --- | --- | --- | --- | --- | --- | --- | --- |
| Cluster | N inds | Mean | SD | Low CI | Upper CI | Mean | SD | Low CI | Upper CI |
| 1 | 1 | 3,68 | 0,07 | 3,54 | 3,83 | 3,68 | 1,37 | 1,00 | 6,37 |
| 2 | 45 | 4,32 | 0,43 | 4,19 | 4,44 | 4,32 | 1,73 | 3,81 | 4,82 |
| 3 | 6 | 3,82 | 0,39 | 3,51 | 4,13 | 3,82 | 1,49 | 2,63 | 5,01 |
| 4 | 18 | 3,95 | 0,41 | 3,76 | 4,15 | 3,95 | 1,56 | 3,24 | 4,67 |
| 5 | 12 | 4,57 | 0,32 | 4,39 | 4,75 | 4,57 | 1,82 | 3,54 | 5,60 |
| 6 | 3 | 3,64 | 0,58 | 2,98 | 4,30 | 3,64 | 1,48 | 1,97 | 5,31 |

# **Supplementary Figures**

**Figure S1.** Analytical precision (difference between two repeated measurements of a same killer whale sample) in relation with peak voltage (height, in mV) as observed in our dataset, leading to discarding δ^15^N-AA values generated from peaks <70 mV.

**Figure S2.** (a) Bulk δ¹⁵N values for killer whales never observed feeding on mammal prey between 2010 and 2023 (*Fish-only*) and for individuals observed feeding on mammals at least in one encounter (*Mixed*). (b) Bulk δ¹⁵N values for the three reference diet groups (see Results) used as a field-validated interpretive framework against which δ¹⁵N-AA-derived hypotheses were tested. In both panels, boxplots show the interquartile range (second and third quartiles), with the horizontal line indicating the median and whiskers representing the first and fourth quartiles. Dots represent individual samples. Stars indicate samples assigned to unexpected clusters in the hierarchical clustering analysis (see Results).

**Figure S3.** Results of the cluster analysis performed on the 27 killer whales observed foraging on mammal prey in addition to fish (mixed diet whales) at least once. Clustering was based on similarities in bulk δ^15^N values and mercury (Hg) concentrations in skin, and and the proportion of encounters involving mammal predation between 2010 and 2023 (MAMM index). Panel (a) shows the two most likely clusters, given the data. Panels (b), (c), and (d) illustrate distinct ecological characteristics of the resulting *Intermediate* and *Top mixed* diet groups, supporting biologically meaningful classifications. In all boxplots, boxes represent the interquartile range, the horizontal line the median, whiskers the first and fourth quartiles, and dots individual observations.

**Figure S4**. (a) Elemental carbon-to-nitrogen (C:N) ratios calculated from the %C and %N in the non-lipid extracted aliquot (see Methods) calculated for each reference diet group (Kruskal-Wallis test: Chi-sq= 1.22, df=2, p=0.54); and (b) correlation between δ^15^N-Phe (blue line), and δ^15^N-Lys (green line) and the C:N ratio, as tentative means to investigate potential influence of diet quality as confounder of dietary signal.

**Figure S5.** (a) Linear regression between the sum of δ¹⁵N-Phe and δ¹⁵N-Lys (δ^15^N**_ΣPhe, Lys_**) and bulk tissue δ¹⁵N, and (b) linear regression between δ^15^N**_ΣPhe, Lys_** and bulk δ^13^C to evaluate potential baseline isotopic variations as confounder of dietary signal in our data.

**Figure S6.** Principal component analysis (PCA) of δ¹⁵N values of amino acids measured in killer whale skin samples. Sex (a) and life-stage groups (b) were included as supplementary qualitative variables and projected post hoc onto the ordination for vizualization. In agreement with redundancy analysis results, no clear separation among sex or life-stage groups was observed, and neither variable was identified as a significant driver of δ¹⁵N-AA variation.

**Figure S7.** (a) Difference in δ^15^N values between the sum of trophic (Glu, Asp, Ala, Leu, Pro) amino acids and the sum of source (Phe, Lys) amino acids (δ^15^N**_Σ_**_Trophic -_ **_Σ_**_Source_) (Kruskal-Wallis rank sum test: hi-squared = 7.7688, df = 2, p-value = 0.02056; Dunn post hoc test: only Fish-only vs. Top mixed was significant: P= 0.016), and (b) δ¹⁵N difference between Glu and Phe (δ^15^N_Glu - Phe_) values, for the three reference diet groups (Kruskal-Wallis test: chi-squared = 9.2977, df = 2, p-value = 0.009573; Dunn posthoc test: only Fish-only vs. Top mixed was significant: P= 0.007). Box depicts second and third quartiles, horizontal line represents the median and whiskers represent the first and fourth quartiles. c) Linear regression between δ^15^N-Thr and δ^15^N_Glu – Phe_.

**Figure S8.** Density plot of (mean) trophic position derived from δ¹⁵N amino acid data (TP_AA_) per season at sampling, showcasing an increase in trophic position from winter to summer.

**References**

Andvik, C., Jourdain, E., Ruus, A., Lyche, J. L., Karoliussen, R., & Borgå, K. (2020). Preying on seals pushes killer whales from Norway above pollution effects thresholds. *Scientific Reports*, *10*(1), 11888.

Gaebler, O. H., Vitti, T. G., & Vukmirovich, R. (1966). Isotope effects in metabolism of 14n and 15n from unlabeled dietary proteins. *Canadian Journal of Biochemistry*, *44*(9), 1249–1257. https://doi.org/10.1139/o66-142

Hoskin, S. O., Gavet, S., Milne, E., & Lobley, G. E. (2001). Does glutamine act as a substrate for transamination reactions in the liver of fed and fasted sheep? *British Journal of Nutrition*, *85*(5), 591–597.

Jourdain, E., Goh, T., Kuningas, S., Similä, T., Vongraven, D., Karoliussen, R., Bisther, A., & Hammond, P. S. (2021). Killer whale (Orcinus orca) population dynamics in response to a period of rapid ecosystem change in the eastern North Atlantic. *Ecology and Evolution*, *11*(23), 17289–17306. https://doi.org/10.1002/ece3.8364

Jourdain, E., Karoliussen, R., de Vos, J., Zakharov, S. E., & Tougard, C. (2020a). Killer whales (Orcinus orca) feeding on lumpfish (Cyclopterus lumpus) in northern Norway. *Marine Mammal Science*, *36*(1), 89–102. <https://doi.org/10.1111/mms.12618>

Jourdain, E., Andvik, C., Karoliussen, R., Ruus, A., Vongraven, D., & Borgå, K. (2020b). Isotopic niche differs between seal and fish-eating killer whales (Orcinus orca) in northern Norway. *Ecology and Evolution*, *10*(9), 4115–4127. https://doi.org/10.1002/ece3.6182

Jourdain, E., Vongraven, D., Bisther, A., & Karoliussen, R. (2017). First longitudinal study of seal-feeding killer whales (Orcinus orca) in Norwegian coastal waters. *PLOS ONE*, *12*(6), e0180099. https://doi.org/10.1371/journal.pone.0180099

Kassambara, A., & Mundt, F. (2020). *factoextra: Extract and Visualize the Results of Multivariate Data Analyses* (Version 1.0.7) [Computer software]. https://cran.r-project.org/web/packages/factoextra/index.html

Lee, K., Jourdain, E., Remili, A., McKinney, M. A., Andvik, C., Fagerlund, C., Karoliussen, R., Borgå, K., & Ruus, A. (n.d.). Integrating fatty acids, stable isotopes, mercury, and predation histories to assess Norwegian killer whale diet. *Marine Ecology Progress Series*.

Nash, A. L., Newsome, S. D., & McMahon, K. W. (2024). On precision and accuracy: A review of the state of compound-specific isotope analysis of amino acids. *Organic Geochemistry*, *195*, 104823.

Olesiuk, P. F., Bigg, M. A., & Ellis, G. M. (1990). *Life history and population dynamics of resident killer whales (Orcinus orca) in the coastal waters of British Columbia and Washington State.* (12; Report of the International Whaling Commission, Special, pp. 209–243).

Olesiuk, P. F., Ellis, G. M., & Ford, J. K. B. (2005). *Life history and population dynamics of northern resident killer whales (Orcinus orca) in British Columbia*. Canadian Science Advisory Secretariat.

Pauly, D., Christensen, V., Dalsgaard, J., Froese, R., & Torres, F. (1998). Fishing Down Marine Food Webs. *Science*, *279*(5352), 860–863. https://doi.org/10.1126/science.279.5352.860

R Core Team. (2018). *R: A language and environment for statistical computing* (Version 4.2.0) [Computer software]. R Foundation for Statistical Computing. https://www.R-project.org/

Ramirez, M. D., Besser, A. C., Newsome, S. D., & McMahon, K. W. (2021). Meta-analysis of primary producer amino acid δ15N values and their influence on trophic position estimation. *Methods in Ecology and Evolution*, *12*(10), 1750–1767. https://doi.org/10.1111/2041-210X.13678

Remili, A., Dietz, R., Sonne, C., Samarra, F. I. P., Rikardsen, A. H., Kettemer, L. E., Ferguson, S. H., Watt, C. A., Matthews, C. J. D., Kiszka, J. J., Jourdain, E., Borgå, K., Ruus, A., Granquist, S. M., Rosing-Asvid, A., & McKinney, M. A. (2023). Quantitative fatty acid signature analysis reveals a high level of dietary specialization in killer whales across the North Atlantic. *Journal of Animal Ecology*, *n/a*(n/a). https://doi.org/10.1111/1365-2656.13920

Rita, D., Borrell, A., Wodarg, D., Víkingsson, G., García-Vernet, R., Aguilar, A., & Loick-Wilde, N. (2024). Amino acid-specific nitrogen stable isotope analysis reveals the trophic behavior of Icelandic fin whales in winter and suggests variable feeding strategies. *Marine Mammal Science*, *40*(3), e13097. https://doi.org/10.1111/mms.13097

Robeck, T. R., Fei, Z., Lu, A. T., Haghani, A., Jourdain, E., Zoller, J. A., Li, C. Z., Steinman, K. J., DiRocco, S., Schmitt, T., Osborn, S., Van Bonn, B., Katsumata, E., Mergl, J., Almunia, J., Rodriguez, M., Haulena, M., Dold, C., & Horvath, S. (2021). Multi-species and multi-tissue methylation clocks for age estimation in toothed whales and dolphins. *Communications Biology*, *4*(1), 1–11. <https://doi.org/10.1038/s42003-021-02179-x>

Ruus, A., Øverjordet, I. B., Braaten, H. F. V., Evenset, A., Christensen, G., Heimstad, E. S., ... & Borgå, K. (2015). Methylmercury biomagnification in an Arctic pelagic food web. *Environmental toxicology and chemistry*, *34*(11), 2636-2643.

Silfer, J. A., Engel, M. H., Macko, S. A., & Jumeau, E. J. (1991). Stable carbon isotope analysis of amino acid enantiomers by conventional isotope ratio mass spectrometry and combined gas chromatography/isotope ratio mass spectrometry. *Analytical Chemistry*, *63*(4), 370–374. https://doi.org/10.1021/ac00004a014

Spiess, A.-N. (2025). *propagate: Propagation of Uncertainty* (Version 1.0-7) [Computer software]. https://cran.r-project.org/web/packages/propagate/index.html

Vongraven, D., & Bisther, A. (2014). Prey switching by killer whales in the north-east Atlantic: Observational evidence and experimental insights. *Journal of the Marine Biological Association of the United Kingdom*, *94*(6), 1357–1365. https://doi.org/10.1017/S0025315413001707
